# Using parenclitic networks on phaeochromocytoma and paraganglioma tumours provides novel insights on global DNA methylation

**DOI:** 10.1038/s41598-024-81486-9

**Published:** 2024-12-02

**Authors:** Dimitria Brempou, Bertille Montibus, Louise Izatt, Cynthia L Andoniadou, Rebecca J Oakey

**Affiliations:** 1 Department of Medical and Molecular Genetics, King’s College London, London, SE1 9RT UK; 2 Department of Clinical Genetics, Guy’s and St Thomas’ NHS Foundation Trust, London, SE1 9RT UK; 3 Centre for Craniofacial and Regenerative Biology, King’s College London, London, SE1 9RT UK

**Keywords:** Classification, Graph theory, DNA methylation, Phenotype, PPGL, Tumour, Cancer, Computational biology and bioinformatics, Genetics, Endocrinology, Oncology

## Abstract

Despite the prevalence of sequencing data in biomedical research, the methylome remains underrepresented. Given the importance of DNA methylation in gene regulation and disease, it is crucial to address the need for reliable differential methylation methods. This work presents a novel, transferable approach for extracting information from DNA methylation data. Our agnostic, graph-based pipeline overcomes the limitations of commonly used differential methylation techniques and addresses the “small n, big k” problem. Pheochromocytoma and Paraganglioma (PPGL) tumours with known genetic aetiologies experience extreme hypermethylation genome wide. To highlight the effectiveness of our method in candidate discovery, we present the first phenotypic classifier of PPGLs based on DNA methylation achieving 0.7 ROC-AUC. Each sample is represented by an optimised parenclitic network, a graph representing the deviation of the sample’s DNA methylation from the expected non-aggressive patterns. By extracting meaningful topological features, the dimensionality and, hence, the risk of overfitting is reduced, and the samples can be classified effectively. By using an explainable classification method, in this case logistic regression, the key CG loci influencing the decision can be identified. Our work provides insights into the molecular signature of aggressive PPGLs and we propose candidates for further research. Our optimised parenclitic network implementation improves the potential utility of DNA methylation data and offers an effective and complete pipeline for studying such datasets.

**Supplementary Information:**

The online version contains supplementary material available at 10.1038/s41598-024-81486-9.

## Introduction

Pheochromocytomas and Paragangliomas (PPGL) are rare neuroendocrine tumours with about 40% of the cases being associated with a heritable germline pathogenic variant in one of the more than 20 susceptible genes^1^. Pheochromocytomas develop in the adrenal medulla, while paragangliomas develop in the extra-adrenal paraganglia. Despite their strong heritability, PPGLs present with notable phenotypic variability, with some probands not developing any tumours and others experiencing aggressive disease progression characterised by distant metastases, positive regional lymph nodes, or local recurrence^2^. Despite two recent classification efforts using whole exome sequencing (WES) and RNA sequencing data^3^, and clinical and biochemical data^4^ respectively, no predictive markers are used for the diagnosis of aggressive PPGL and the molecular mechanisms behind this phenotype are poorly understood. This limits the development of personalised monitoring and treatment plans. PPGL patients are being regularly monitored through biochemical tests and imaging even after complete removal of the tumour^5^. Unfortunately, for patients with aggressive PPGL there are limited treatment options, and the outcomes are often poor^6^.

DNA methylation is an epigenetic modification influencing the regulation of gene expression. More specifically, DNA methylation is the addition of a methyl group to the cytosine, mostly occurring at the CpG dinucleotides in the mammalian genome, i.e., genomic locations where a cytosine nucleotide is followed by a guanine nucleotide. The presence of DNA methylation can silence the expression of a gene by eliminating the binding of transcription factors in the gene promoter^7^. DNA methylation is considered a cancer hallmark and disturbances are common in cancer^8^. DNA methylation arrays quantify the DNA methylation state at CpG sites and are valuable for decoding the functional role of DNA methylation. Understanding the DNA methylation patterns of PPGLs could lead to more effective management of the disease.

PPGLs are separated into 3 clusters based on the associated pathogenic variant. The clustering also reflects their transcriptomic properties. Cluster 1 is characterised by pseudohypoxia and is further divided into two subclusters, Cluster 1 A including pathogenic variants affecting TCA cycle related genes and Cluster 1B including pathogenic variants in VHL and EPAS1. Clusters 2 and 3 include pathogenic variants affecting WNT signalling and kinase signalling respectively^2^.

Cluster 1 A tumours are particularly interesting due to their methylation patterns and aggressive progression potential. More specifically, cluster 1 A is characterised by global hypermethylation^2^and PPGLs associated with pathogenic variants in gene SDHB, which belong to cluster 1 A, have been associated with higher rates of aggressive phenotype^9^. Studying the DNA methylation differences between aggressive and non-aggressive PPGL tumours can shed light on the molecular mechanism of the phenotypic variability and assist the discovery of predictive markers with clinical application. However, the complexity of the DNA methylation data in combination with the rarity of this cancer have decelerated the process of decoding the DNA methylation signature of aggressive PPGLs.

The resolution of the DNA methylation quantification arrays has improved rapidly in the last 10 years starting from Infinium HumanMethylation27 arrays, assaying 27,578 CpG dinucleotides, and reaching Infinium MethylationEPIC v2.0 arrays, including over 935,000 CpG sites, with the most recent technology. However, array normalisation methods lack reproducibility, especially for probes with small variance^10^. Differential methylation methods are still underrepresented in the literature leading to the lack of a standardised pipeline. Additionally, most of the commonly used techniques, such as bump hunting^11^ and statistical F-test, when applied on our data, were proven to be sensitive to pre-processing choices and their results presented little overlap.

More recent publications have achieved remarkable results in the exploration of DNA methylation data by employing machine learning^12,13^. Such methods, though insightful, offer limited possibilities for smaller datasets with a large list of attributes often encountered in biomedical research. The problem of “small n, big k”, i.e., small number of samples (n) with many attributes (k), is prevalent in genomic research and in the study of rare diseases. Overcoming this issue is essential to avoid overfitting and draw reliable conclusions.

Parenclitic networks^14^ proposed by Zanin et al. offer a more elaborate approach to extract valuable information from biological data, including DNA methylation arrays, and to overcome the “small n, big k” problem. A parenclitic network, in the context of this work, is a graph representing the deviation of a sample from the expected non-aggressive behaviour. Using this method, each sample is represented by a graph. This allows for topological features selection as descriptors of the methylation state of each sample and decreases the dimensionality of the problem. As opposed to other methods, which assume correlation between the genomic location of the CG sites and their interaction, parenclitic networks follow an agnostic approach considering interactions between CG sites regardless of their genomic position.

Here, we apply parenclitic networks to predict the aggressive phenotype of PPGL based on their DNA methylation state. For the development and evaluation of the classifier we utilise two separate PPGL datasets available in the public domain and their clinical attributes. More specifically, The Cancer Genome Atlas (TCGA) dataset^2^ has been used for modelling the non-aggressive phenotype as well as training of the classifier, while a dataset published in Array Express^15^ has been used for the evaluation of the model. This ensures the reliability of the classifier’s performance by minimising data bias and proves its applicability in real world scenarios. Our classifier achieved 70% balanced accuracy and 0.7 area under the receiver operating characteristic curve (ROC-AUC), demonstrating for the first time that there are differences in the DNA methylation patterns between aggressive and non-aggressive PPGL and highlighting their predictive potential. Moreover, the CG loci with high absolute value coefficients in the logistic regression classifier are biologically relevant, with some being previously associated with enhancer regions, transcription factor binding sites and marker genes of aggressive progression in other cancers. These findings are a first step towards personalised treatment for PPGL patients.

## Data description

We used two publicly available DNA methylation datasets from PPGL tumour tissue. The 450 K array data are published in The Cancer Genome Atlas (TCGA) (dbGaP Study Accession: phs000178)^2^ and the 850 K array data are published in Array Express (AE) (Study Accession: E-MTAB-13433)^15^. Both datasets provide phenotypic information about the samples. Aggressive disease is defined by having distant metastasis, positive local lymph nodes, or local recurrence (non-metastatic) tumours. PPGLs stem from chromaffin cells or their progenitors, hence, distant metastases occur in locations with no chromaffin tissue, i.e. bone, liver or lung, as per World Health Organisation (WHO) definition^16^.

The TCGA data contained 173 samples, 157 of which are non-aggressive and 16 are aggressive cases. The AE dataset contains 34 samples, out of which 20 are non-aggressive and 14 are aggressive. The AE dataset has higher resolution using 850 K arrays compared to the TCGA dataset, which uses 450 K arrays. 850 K arrays are an enrichment of 450 K arrays to include more CpG sites in enhancer regions and have been shown to achieve high reproducibility on the 450 K array CpG sites^17^.

From the TCGA dataset, 141 randomly selected non-aggressive samples have been used for modelling, the remaining 16 non-aggressive samples and all 16 aggressive samples have been used for training. The modelling and training datasets are non-overlapping. The entire AE dataset has been used for evaluation.

## Results

### DM analysis

**Fig. 1:**
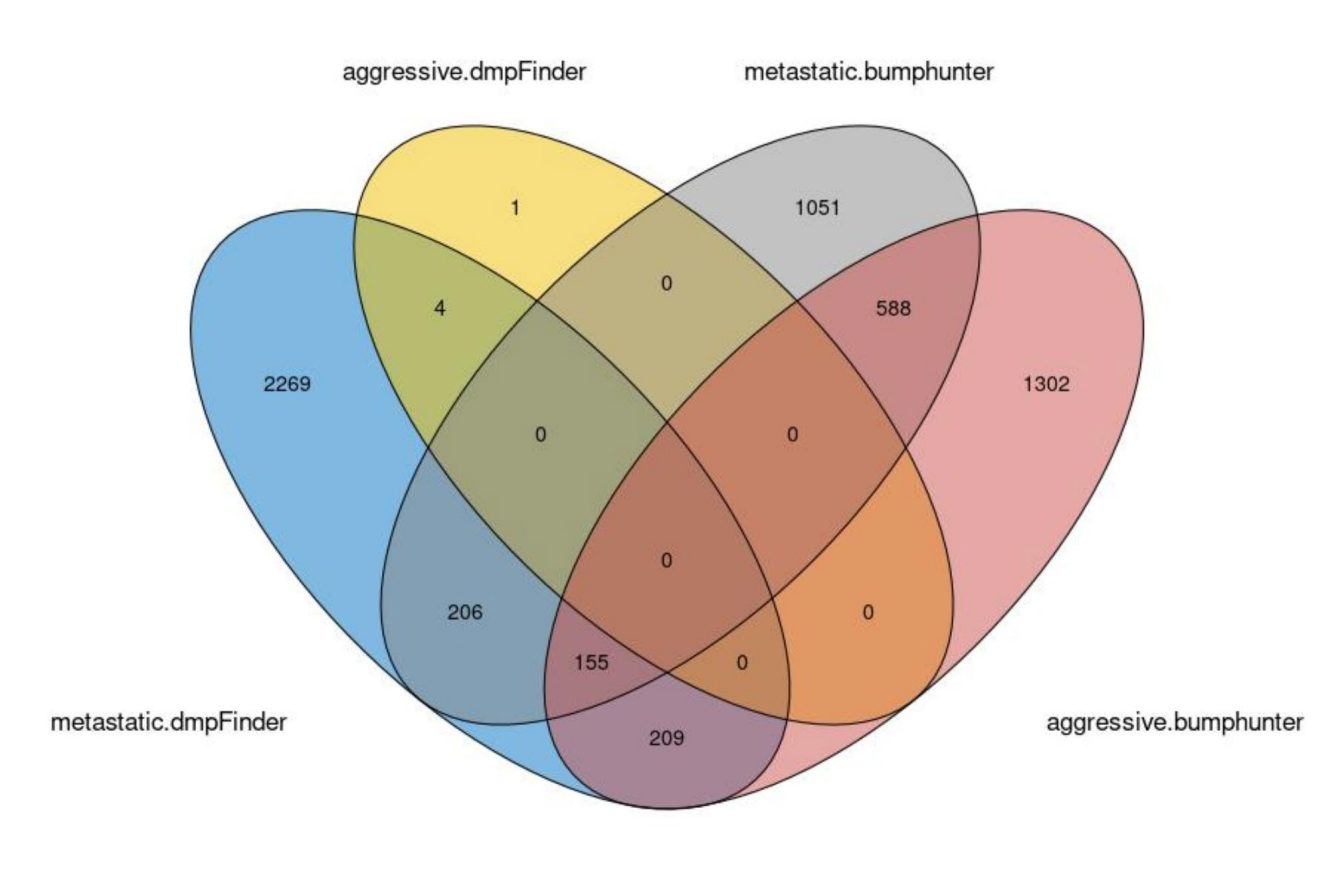
Venn diagram presenting the overlap in the genes associated with differentially methylated loci and regions using two different methods, dmpFinder and bumphunter. The two methods have been applied to identify differentially methylated CG loci or regions respectively between aggressive and non-aggressive, and metastatic and non-metastatic PPGL cases. The first comparison is denoted with “aggressive” and the latter with “metastatic”. When comparing aggressive to non-aggressive the results of the two methods show no overlap. Also when comparing metastatic to non-metastatic the overlap is small. In addition, the two comparisons for the same method showed limited overlap despite the large overlap of the metastatic and aggressive cases.

Differential DNA methylation analysis was performed using the AE dataset and all loci satisfying the quality control criteria were included with no filtering for specific function or properties. DmpFinder detected 9 significantly differentially methylated CG loci between aggressive and non-aggressive PPGLs, while bumphunter detected 905 bumps. The results of the two methods, translated into associated genes to allow for comparison between CG sites and bumps, presented no overlap (Fig. 1). This difference can be partly explained by the algorithmic differences between dmpFinder and bumphunter. The first is testing for differential methylation on a specific genomic position, while the latter considers wider genomic regions. However, using the same method to compare aggressive to non-aggressive or metastatic to non-metastatic also showed small overlap despite the big overlap in the aggressive and metastatic cases.

The lack of overlap between the methods as well as the inconsistency are not surprising given that bumphunter has been found to have high false positive rates ranging from 35–95%^18^, and the F-test, performed by dmpFinder, assumes normally distributed populations, which is not necessarily true. This underlines the need for a different approach in analysis DNA methylation data.

#### Parenclitic networks

**Fig. 2:**
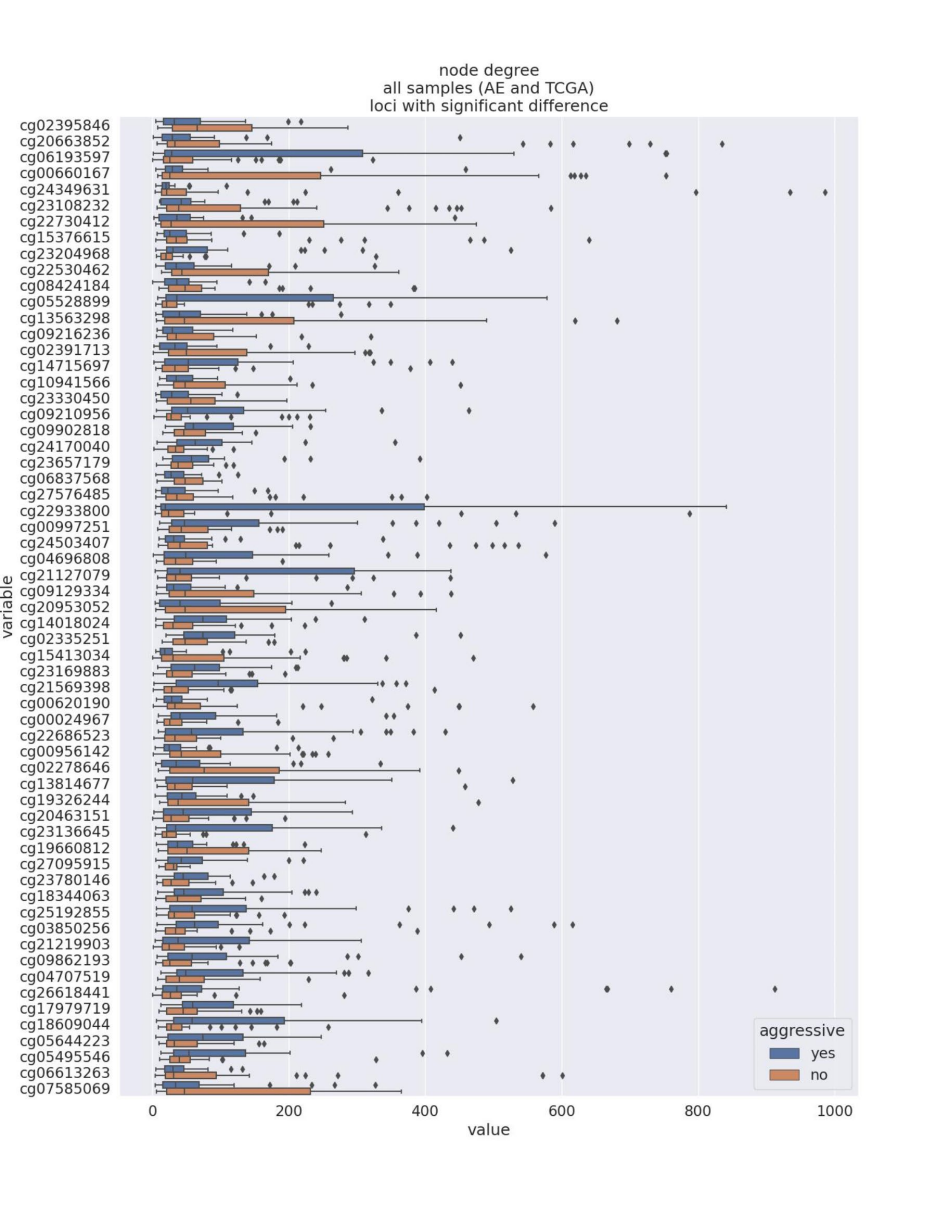
Boxplot of the node degree of aggressive (in blue) and non-aggressive (in orange) samples per CG locus for all samples (AE and TCGA). The displayed CG loci show significant difference in the node degree between aggressive and non-aggressive samples. This visualisation demonstrates the differences in the parenclitic networks’ structure between aggressive and non-aggressive tumours.

We used parenclitic networks to represent the DNA methylation patterns of PPGL tumours and study perturbations in aggressive cases. Evident differences in the structure of the parenclitic networks between aggressive and non-aggressive tumours are observed and illustrated in the topological features. By plotting the node degree at the loci with significant differences in node degree between aggressive and non-aggressive AE and TCGA data (excluding the samples used for modelling), distinct patterns emerge for aggressive and non-aggressive cases (Fig. 2). Similar differences are observed for other topological features, such as degree centrality and betweenness centrality (Figure S1, Figure S2). This provides evidence of perturbation in the DNA methylation between aggressive and non-aggressive PPGL.

#### Classification

**Fig. 3:**
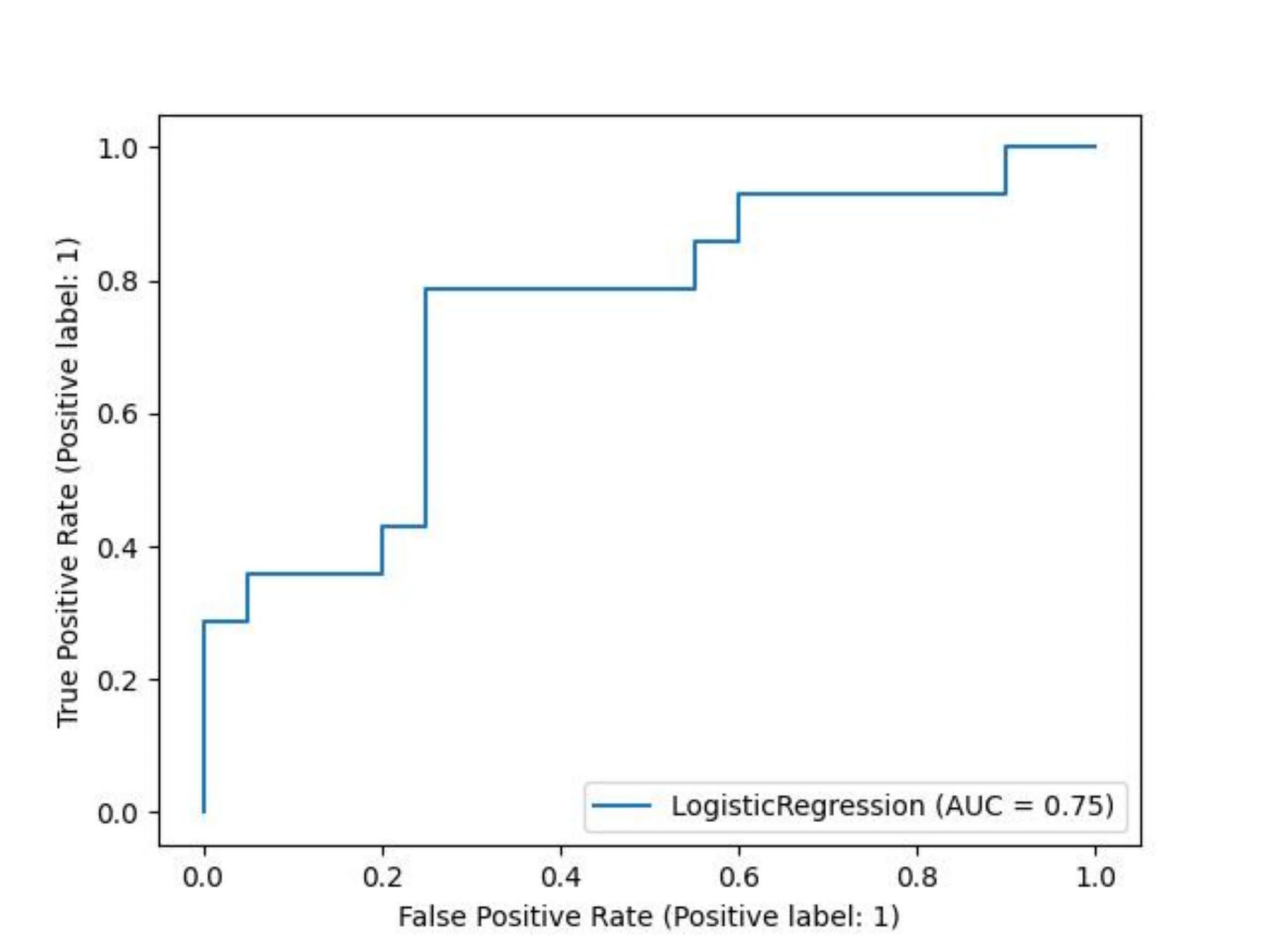
ROC curve of the logistic regression classifier based on the node degree of their parenclitic network.

A logistic regression classifier was implemented to classify samples as aggressive or non-aggressive based on the node degree of their parenclitic network. The classifier achieved 71% accuracy, 70% balanced accuracy, 0.64 F1-score and 0.7 ROC-AUC as illustrated in Fig. 3. This result was consistent for different random state choices indicating convergence to the global optimum. Given the fact that the train and test samples are from independent datasets, assayed using different technologies, namely 450 K and 850 K arrays, and considering the minimal pre-processing and quality restrictions implemented, the classification results are expected to be replicable and reproducible when applied on different datasets. Moreover, the loci having coefficients with larger absolute value present biological relevance. This is particularly important as it highlights the interpretability of our approach and provides further evidence that the classification is based on the underlying molecular mechanism of aggressive disease progression.

#### Candidates

**Fig. 4:**
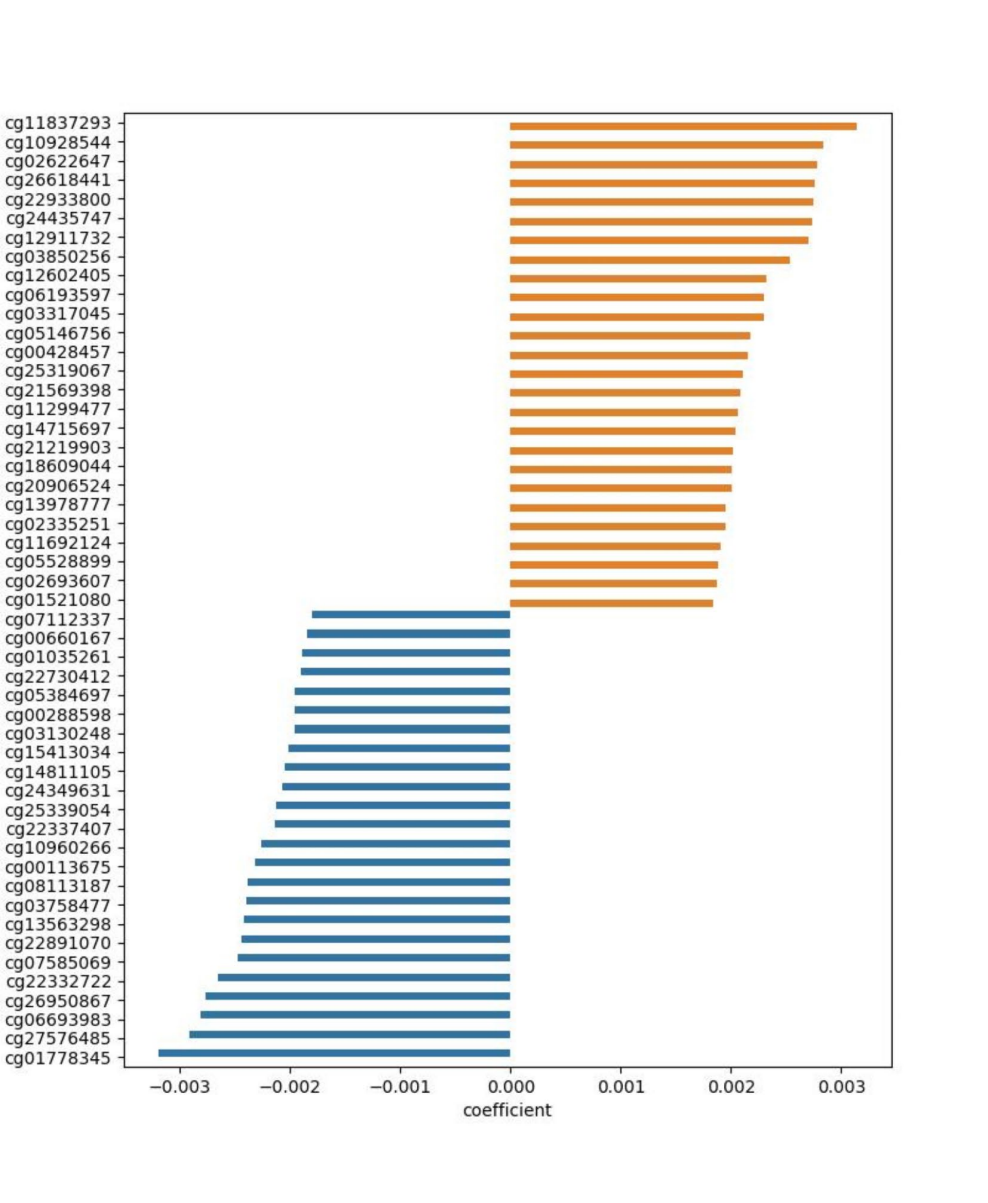
Barchart of the logistic regression coefficient values for the CG loci with absolute coefficient value in the top 5%. The blue bars represent positive coefficient values, i.e. the node degree of the respective CG locus increases the probability for the sample to be classified as aggressive. The orange bars represent negative coefficient values, i.e. reduce the probability of classification as aggressive.

Amongst the top 5% of CG loci with the highest logistic regression coefficients absolute values were some of remarkable biological relevance, which have been identified as potential markers of aggressive disease with molecular significance and are worth investigating further. Out of the 50 loci with the highest influence, 33 are associated with a gene, 14 are located in an enhancer region and 10 are located in transcription factor binding sites according to the methylation array annotation. The loci with coefficients in the top 5% are illustrated in Fig. 4 and their coefficients are listed in Table S4. The full list of CG loci and their logistic regression coefficients values are presented in Table S6.

Among the CpG loci increasing the probability of classification as aggressive are cg10928544, cg22933800, cg24435747 and cg05146756 (Table 1). cg10928544 is intragenic to *CCDC88C*, a negative regulator of the WNT pathway with prognostic relevance for cutaneous melanoma^19^. cg22933800 is located on a POLR2A binding site close to the promoter of *HLA-DQA1*, high expression of which predicts poor outcomes of oesophageal squamous cell carcinoma^20^. cg24435747 is close to the promoter of *IFNLR1*, which has been found to play a role in the pathogenesis of pancreatic ductal adenocarcinoma^21^and diagnosis of early pancreatic cancer^22^. Lastly, cg05146756 is located close to the promoter of *MIR31HG*, which regulates proliferation and migration in breast cancer and gastric cancer cells^23,24^, contributes to hepatocellular carcinoma progression^25^and is a prognostic predictor for poor outcomes in thyroid cancer^26^and for malignant cancer^27^.

In contrast, cg27576485, cg26950867, cg22332722, cg22891070, cg13563298 and cg03758477 are found to decrease the probability of a sample classifying as aggressive (Table 1). cg27576485 is located on a transcription factor binding site and is intragenic to *PTRF*, which plays a role in glioma^28,29^, controls prostate cancer metastasis^30^, and suppresses progression of colorectal cancers^31^. cg26950867 is intragenic to *FN1*, close to the promoter, on a POLR2A binding site. *FN1*is an extracellular matrix marker for cancer^32^, associated with poor survival in oesophageal squamous cell carcinoma^33^and a potential marker of papillary thyroid cancer^34^. cg22332722 is intragenic to *CDH2*, located close to the promoter. *CDH2*inhibits cholangiocarcinoma^35^, mediates the migration of bone-marrow derived mesenchymal stem cells towards breast cancer cells^36^and assists early diagnosis of invasion in ductal carcinoma^37^. cg22891070, is located in a CTCF binding site and is intragenic to the hypoxia inducible factor *HIF3A* potentially reflecting the connection between the pseudo-hypoxic PPGL cluster and aggressive progression. cg13563298 is intragenic to *WNK2*, a tumour suppressor, and lastly, cg03758477 is intragenic to *SEC14L1*found to be a prognostic factor in breast cancer^38^and is also associated with endometrial serous carcinoma^39^and prostate cancer^40^. The genomic location of each of these CG loci has been depicted using the University of California Santa Cruz (UCSC) Genome Browser^41,42^ and can be found in File S3.

**Fig. 5:**
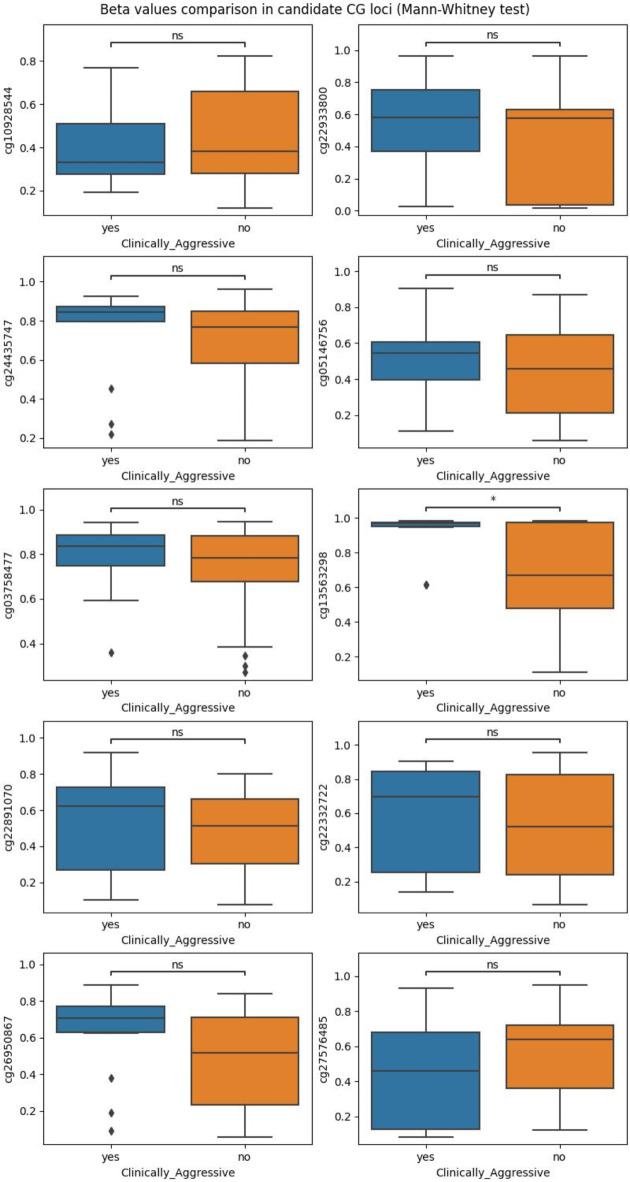
Boxplots of the beta values for aggressive (in blue) and non-aggressive (in orange) samples on the candidate CG loci. The differences between aggressive and non-aggressive per CG locus have been tested using the Mann-Whitney statistical test and the significance is indicated in the top of each plot, with “ns” implying no significant differences in the beta values between aggressive and non-aggressive and * indicating a p-value less than 0.05. Only one locus displays significant differences. This is expected because the parenclitic network approach identifies CG loci, variation in which is likely to have a widespread effect in the methylation, instead of CG loci with locus-specific differences in DNA methylation.

**Fig. 6:**
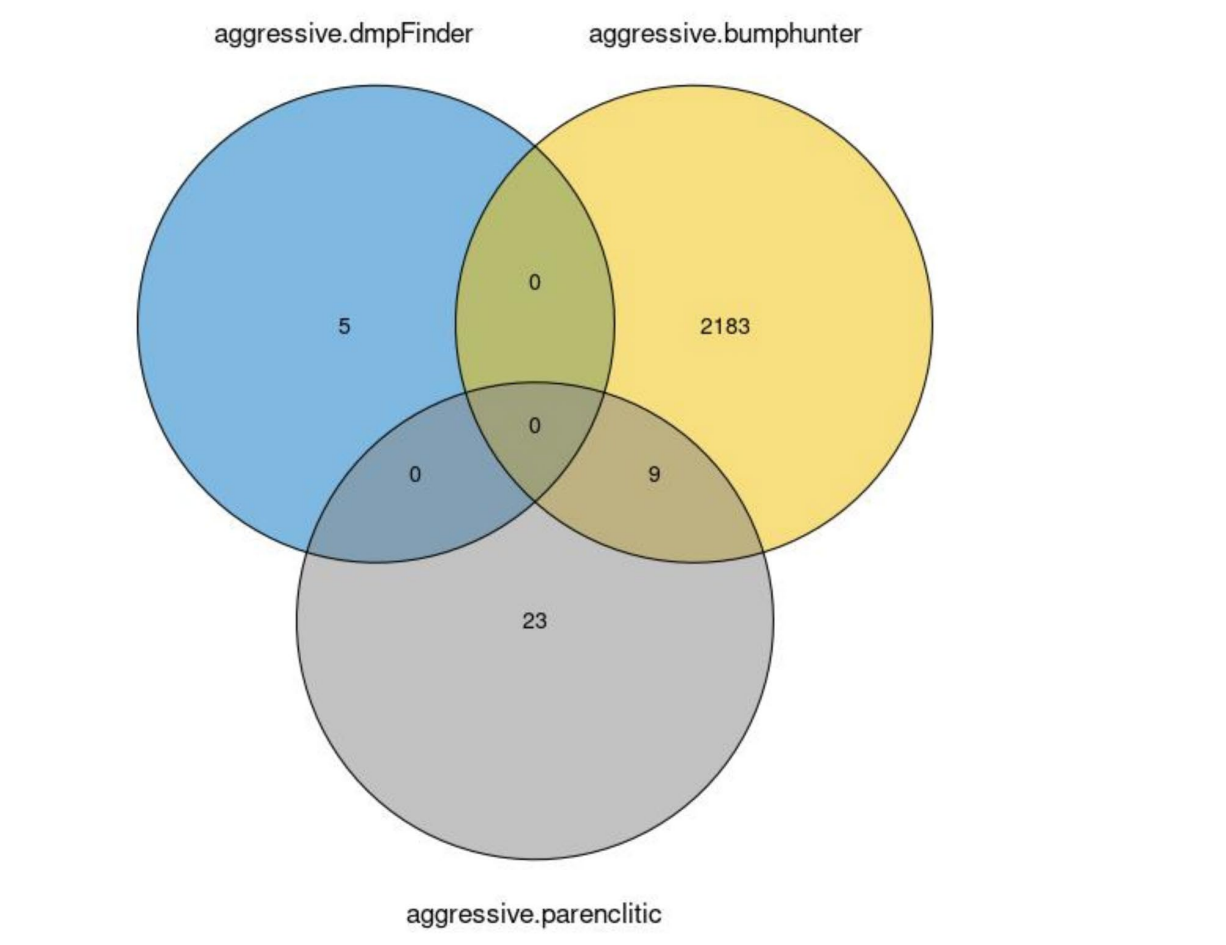
Venn diagram presenting the overlap in the genes associated with differentially methylated loci and regions identified by dmpFinder and bumphunter respectively and candidates identified using parenclitic networks. All three methods have been applied to identify differences between aggressive and non-aggressive PPGL. There is no overlap between dmpFinder and the other methods. There is a small overlap between bumphunter and Parenclitic networks including KIAA0040, ADAMTS12, ELOVL7, OCA2, KIF26B, VGLL4, HLA-H, HIF3A, GDAP2. Bumphunter presents greater similarity to the parenclitic networks approach presented here compared to dmpFinder. This is because dmpFinder investigates differences at the level of individual CG loci, whereas bumphunter is looking at broader regions. Parenclitic networks broaden the area of interest further by taking into account interaction between loci regardless of genomic distance. Parenclitic networks, in contrast to dmpFinder and bumphunter, identify CG loci that play a key role in the DNA methylation alterations in multiple regions of the genome instead of isolated changes.

The beta values on these loci are not found to be significantly different between aggressive and non-aggressive loci (except for cg13563298) (Fig. 5). There is limited overlap with the differentially methylated sites and regions identified using dmpFinder and bumphunter (Fig. 6). In particular, there is no overlap between the results of dmpFinder and parenclitic networks. There are 9 genes identified by both bumphunter and parenclitic networks, namely KIAA0040, ADAMTS12, ELOVL7, OCA2, KIF26B, VGLL4, HLA-H, HIF3A, GDAP2. This overlap further supports the importance of incorporating relationships between CG loci in the analysis of DNA methylation data. Unlike more traditional methods, parenclitic networks offer the opportunity to discover more complex relationships and perturbation in the data, instead of focusing on isolated differences, while providing meaningful molecular insights.

**Table 1:** **This table summarises for each candidate CG locus its logistic regression coefficient and its role in the classification, the associated gene according to UCSC and any associations with transcription factor binding sites (TFBS) according to UCSC**

CG locus	Coefficient	Classification as aggressive	Gene	TFBS
cg10928544	0.0028	increases probability	CCDC88C	
cg22933800	0.0027	increases probability	HLA-DQA1	GM12878 POLR2A, GM12878 POLR2A-4H8
cg24435747	0.0027	increases probability	IFNLR1	
cg05146756	0.0027	increases probability	MIR31HG	
cg03758477	-0.0024	decreases probability	SEC14L1	
cg13563298	-0.0024	decreases probability	WNK2	
cg22891070	-0.0024	decreases probability	HIF3A	Ovary CTCF 1, K562 CTCF 1, K562 CTCF t
cg22891070	-0.0027	decreases probability	CDH2	
cg26950867	-0.0027	decreases probability	FN1	HepG2 POLR2A h
cg27576485	-0.0029	decreases probability	PTRF	neuralCell SMC3, K562 IKZF1 1, H1-hESC YY1, IMR90 CEBPB

## Discussion

This work offers new insights into the molecular landscape of PPGL using advanced methods with promising outlook in the field of biomedical research. The role of the methylome in disease in general and in the development of these tumours in particular is still poorly understood and research in this field is essential in order to unlock the full potential of such datasets. This work is a step towards understanding the epigenetic drivers of aggressive PPGL and developing personalised treatment and monitoring options to improve patient outcomes.

Although it is known that PPGL caused by pathogenic variants on *SDHB* are the most likely to progress into aggressive disease and these tumours have been associated with global hypermethylation, the impact of this methylation pattern as well as potential drivers of this behaviour remain unknown. This work highlights the role of DNA methylation perturbations in aggressive PPGL and identifies candidates. Biological validation of the findings, however, is essential to fully understand the effect of these DNA methylation changes in gene expression and evaluate their clinical relevance.

Common differential methylation analysis techniques fail to identify widespread but subtler DNA methylation changes and to consider interactions between distant CG loci. This has hindered scientific advancement with regards to the methylomes of tumours and, in particular, also PPGL research. Using parenclitic networks we have overcome some of the limitations of traditional methods and have unlocked new insights into a potentially more complex molecular mechanism behind the phenotypic variability of PPGL tumours.

Graph based approaches are becoming increasingly popular in the study of DNA methylation. Weighted Gene Co-Expression Network Analysis (WGCNA)^43^, a network analysis method to identify groups of co-expressed genes, has been extended to methylome applications^44^. In WGCNA, the networks are built based on correlation, whereas in parenclitic networks the baseline is described by a model and the networks are built based on deviation from this model. While both methods provide mechanistic insights, WCGNA focuses on identifying groups of co-methylated loci, whereas parenclitic networks reveal differences between two groups of samples, in this case aggressive and non-aggressive PPGL. Graph-based machine learning approaches are also utilised in DNA methylation research, more recently in the context of multi-omics^45–48^. Such methods are very powerful, but due to increased data needs their application in the study of rare disease is limited.

Parenclitic networks provide biological insights, perform well on smaller datasets as well as larger ones, and can be used for a wide range of data. Moreover, they are capable of revealing complex relationships hidden in the data agnostically. These characteristics make them particularly suitable for biomedical research and rare diseases. In combination with the classifier, the presented pipeline can be used for a wide range of research applications and support the advancement of the field.

Despite the applicability of parenclitic networks in a wide range of questions, the computational cost poses an important barrier to this method. Modelling the behaviour of each pair of genomic regions and calculating the distance of each new observation for each of these pairs is computationally expensive. Due to this cost and the limited computational resources available, in this work only the top 1000 most variant loci have been taken into consideration when creating the networks. This massively compromises the resolution of the dataset and excludes potentially valuable loci from further analysis.

Successful classification of patients’ samples as aggressive and non-aggressive based solely on DNA methylation is a breakthrough in the study of the tumours’ methylome. Even though phenotype classification approaches for candidate discovery have the potential of achieving better performance, considering the data limitations of a rare disease and the methodological limitations of the underrepresented methylome data, achieving balanced accuracy of 70% and ROC-AUC 0.7, clearly indicates substantial differences in the methylome of aggressive and non-aggressive PPGL. Most importantly, the robustness of the classifier has been tested and showed consistency for various random states and against different evaluation metrics, further enhancing the reliability of this result. It is worth mentioning, that phenotypic classifiers based on the DNA methylation of the discovered candidates perform significantly better achieving (Table S9), which further demonstrates the role of DNA methylation in PPGL phenotypic variability and highlights its diagnostic potential.

Overall, this approach provides a point of reference for investigations into the PPGL methylome, it promotes our understanding of its involvement in the aggressive phenotype and provides potential candidate differentially methylated regions to be validated empirically. Given the lack of predictive phenotypic markers for PPGL, understanding the molecular mechanism(s) driving phenotypic heterogeneity between patients as well as identifying novel markers is necessary for improving patient care and outcomes.

The pipeline presented is transferable, can support various application, especially in biomedical research, and has the potential to encourage higher quality research results using DNA methylation data. The methylome is currently underrepresented compared to other omics and the available methods for extracting biological insights are limited. Hence, the published pipeline fills a gap in available computational options for methylation research and provides a novel tool for extracting meaningful information from such datasets.

Regarding PPGL research, this analysis highlights the role of the DNA methylation perturbations in the phenotype and encourages further study of the tumour methylome. Future research is necessary to validate the identified candidates. Moreover, our classifier as a whole or the highlighted features selectively can be incorporated in other phenotypic classifiers of the disease to boost performance and further strengthen the reliability of the results. The optimisation of such approaches plays an important role in accelerating biomedical discovery and supporting the development of clinical predictors.

## Methods

### Data processing

The data processing has been minimal and limited to the essential steps to ensure real-world applicability of the findings. A pipeline following the one presented by James E. Barrett et al.^49^ was used for quality control and normalisation of the samples. Probes with detection p-value less than 0.05 were removed. Samples with more than 10% failed probes were removed. Probes containing SNPs, located in non-CpG regions or in the sex chromosomes were filtered. The normal exponential out-of-band (noob) method^50^ was used for background correction and dye-bias normalisation. The beta-mixture quantile dilation (BMIQ)^51^ method was used for design bias correction. The DNA methylation levels are expressed by the beta value, which represents the methylation ratio per locus and takes values between 0 and 1, with 0 representing unmethylated state and 1 methylated state^52^.

#### Differential methylation analysis

To understand the DNA methylation differences between aggressive and non-aggressive samples differential methylation analysis of the data was performed. Two different methods were used, and the results were compared. The *dmpFinder* function from the *minfi *package^53^ in R^54^ (version 4.2.1) was used to understand the differences between aggressive and non-aggressive samples at each locus. This method performs F-test on each locus and identifies those with significant difference. We also applied the bump hunting method^11^ to identify region-wide deviation rather than locus-specific differences. To compare the results, we assigned the identified differentially methylated loci and bumps to genes using the UCSC reference genome (Genome Reference Consortium GRCh37) from minfi’s getAnnotation function and the matchGenes function from the bumphunter package respectively.

#### Parenclitic networks

**Fig. 7:**
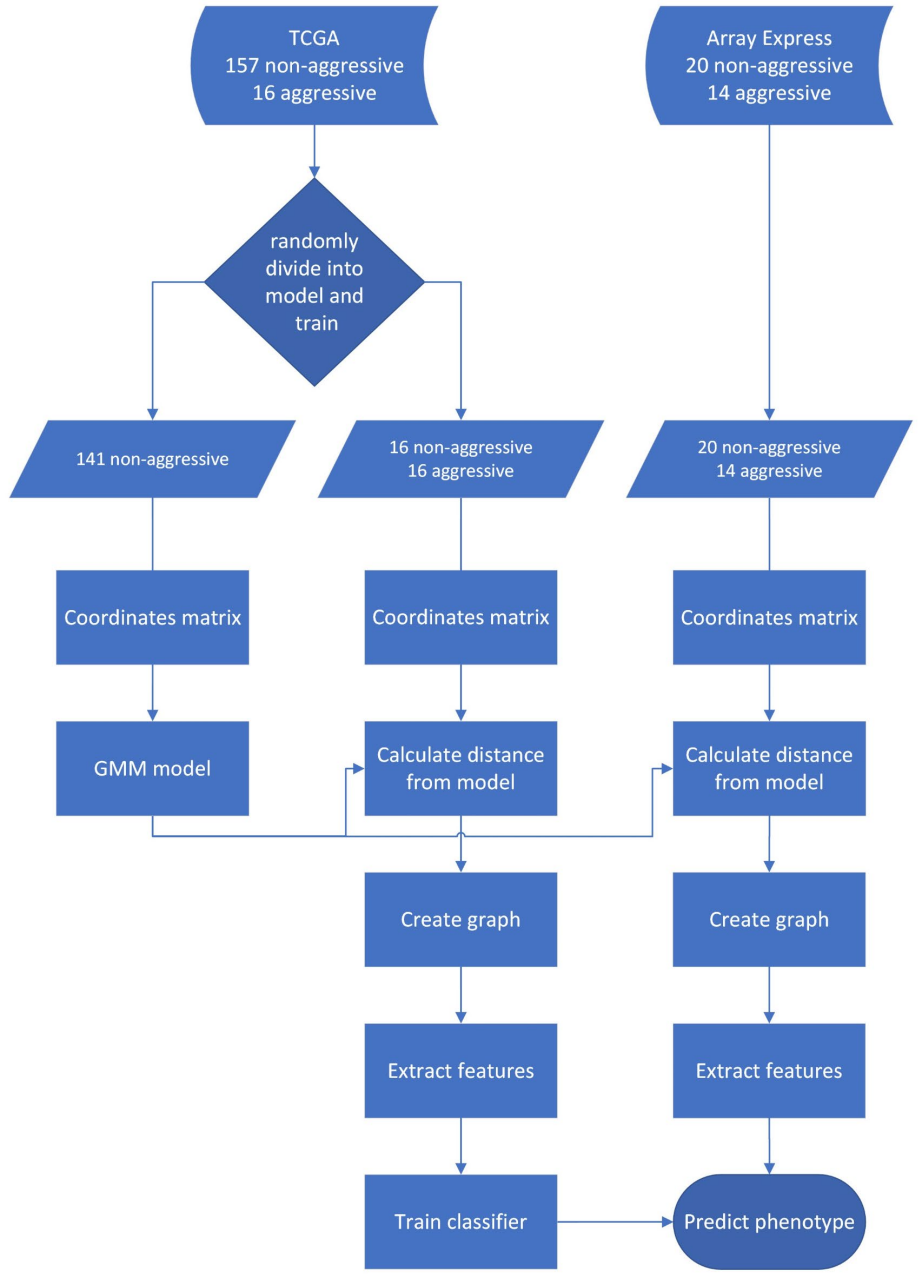
This flowchart schematic represents the pipeline, including the model, training - testing data split, the parenclitic network implementation and the classification.

For the construction of the parenclitic network, the top 1000 most variable methylation loci have been selected from the TCGA cohort due to limitations of the available computational resources. This choice is made based on the low reproducibility of low variability DNA methylation probes^10^. These 1000 loci are included in both 450 K and 850 K arrays and, hence, in both datasets. For each sample, from the TCGA and AE datasets, the coordinates matrix was created using the beta values of each pair of loci. For the modelling of the non-aggressive samples, we used all the non-aggressive samples from the TCGA except 16 randomly selected, which were used together with the 16 aggressive samples from TCGA cohort for training the classifier. For each pair of loci a Gaussian Mixture Model (GMM) with up to 4 components was fitted. For each sample in the train or test dataset a parenclitic network was constructed. Each methylation locus is represented by a node, the edges are weighted by the Mahalanobis distance^55^ of the observed coordinates for each pair of loci from the closest component of the GMM. An outline of this process is presented in Fig. 7 as part of the complete pipeline.

The implementation of the parenclitic networks followed the structure of the initially presented method^14^ with a few adjustments to optimise it for DNA methylation data. These adjustments are presented for the first time as part of this paper and optimise the application of parenclitic networks on DNA methylation data. More specifically, using a GMM for modelling the behaviour of non-aggressive tumours instead of logistic regression reflects on the nature of DNA methylation beta values. When a locus is methylated or unmethylated the beta values are close to 1 or 0 respectively with little variation due to measuring errors. There are 4 distinct methylation states of interest when comparing aggressive and non-aggressive PPGL, namely both methylated, both unmethylated, or one methylated and one unmethylated. The case, that all 4 statuses are present simultaneously, as well as all possible combinations of these 4 statuses, can be represented using a GMM with 4 components. Cases of intermediate methylation status, i.e., beta values which are not clustering around 0 or 1, are also appropriately represented by the GMM. Choosing a mixture of up to 4 components, allows for inter-sample variability as well as inclusion of loci with intermediate methylation status, while still fitting the data well. The Mahalanobis distance^55^ was used to calculate the distance of each sample from the nearest Gaussian mean.

For the construction of the parenclitic networks, the set of nodes is defined as

$$\:\text{V}\:=\:\left\{\text{c}\text{g}\right|\:\text{c}\text{g}\:\text{a}\:\text{C}\text{p}\text{G}\:\text{s}\text{i}\text{t}\text{e}\:\text{o}\text{n}\:\text{t}\text{h}\text{e}\:\text{a}\text{r}\text{r}\text{a}\text{y}\}$$

the set of weighted edges is defined for each sample as

$$\:\text{E}\:=\:\left\{\left({\text{c}\text{g}}_{\text{i}},{\text{c}\text{g}}_{\text{j}}\right)\right|{\text{c}\text{g}}_{\text{i}},\:{\text{c}\text{g}}_{\text{j}}\:\text{d}\text{i}\text{s}\text{c}\text{t}\text{i}\text{n}\text{c}\text{t}\:\text{e}\text{l}\text{e}\text{m}\text{e}\text{n}\text{t}\text{s}\:\text{i}\text{n}\:\text{N}\}\:\:\:$$

and the weight of $\:\left({\text{c}\text{g}}_{\text{i}},{\text{c}\text{g}}_{\text{j}}\right)$ is defined as

$$\:w(c{g}_{i},c{g}_{j})=\underset{\text{n}}{\text{min}}{d}_{M}(\left({\beta\:}_{c{g}_{i}},{\beta\:}_{c{g}_{j}}\right),\left(\:{\mu\:}_{{G}_{1}^{n}},{\mu\:}_{{G}_{2}^{n}})\right),$$

where $\:{d}_{M}$ is the Mahalanobis distance, $\:{\beta\:}_{c{g}_{i}}$, $\:{\beta\:}_{c{g}_{j}}$ the observed beta values at $\:c{g}_{i}$and $\:c{g}_{j}$ and ($\:{\mu\:}_{{G}_{1}^{n}}$, $\:{\mu\:}_{{G}_{2}^{n}}$) the mean of the n-th component of the GMM model.

#### Topological features

The topological features are descriptors of the networks architecture and provide meaningful insights into its structure. There is a wide range of features that are used in graph-based machine learning implementations^56^, a subset of which were applied in this work. The node degree is the sum of the weighted edges connected to a node. The eigenvector centrality represents the transitive influence of a node^57,58^. Betweenness centrality is the sum of the fraction of all-pairs shortest paths that pass through a node^59,60^. Degree centrality is the fraction of nodes a node is connected to. Second order centrality is the standard deviation of the return times to a node of a perpetual random walk on the graph^61^. Eccentricity is the maximum distance from the node to all other nodes^62^. The formal definitions can be found in Supplementary Methods S8.

Different topological features were extracted from the parenclitic networks and were evaluated for their classification potential. The extracted features included descriptors of the nodes connectivity and influence, i.e., node degree, eigenvector centrality, betweenness centrality, degree centrality, second order centrality, and descriptors of the overall graph topology, i.e., graph eccentricity. This offered an extensive representation of the graphs’ properties and structure. All topological features values for all samples can be found in Table S7. Node degree was found to outperform other topological features and reflected the differences between aggressive and non-aggressive samples. Therefore, the node degree was selected as input for the classification.

Topological features are a mathematical way of expressing interactions between CG loci and understand complex epigenetic relationships. Features describing the connectivity of a CG locus underscore the importance of this locus in influencing DNA methylation alterations. Better-connected loci are more likely to play a key role in shaping the epigenetic landscape. The overall topology, represented by the graph eccentricity, is also interesting to study. Changes in the general structure of the network highlight pivotal DNA methylation perturbations in the sample.

#### Classification

The classifier received as input a topological feature describing the sample’s parenclitic network and performed binary classification of the sample as aggressive or non-aggressive. The classification performance of different classifiers, i.e. logistic regression, k Nearest Neighbours, decision trees, neural networks, AdaBoost, bagging of k Nearest Neighbours and bagging of logistic regression, and topological features has been assessed and the results can be found in Table S5. Logistic regression^63^ on the node degree was chosen as the final classifier based on performance, interpretability and stability. More specifically, logistic regression outperformed the other classifiers in most cases and showed consistent and reliable performance regardless of the choice of random state. It is a statistical technique and as such more intuitive and interpretable compared to other black box machine learning classifiers, e.g. neural networks. The classifier was trained on TCGA samples not seen in the modelling process and evaluated on the independent AE dataset.

It is worth clarifying that classification was applied in this work in the scope of candidates’ discovery and is, therefore, inherently different to classification based on the identified candidates of the different methods, i.e., bumphunter, dmpFinder and Parenclitic networks. For clarity, the performance of classification based on the loci highlighted by each method is presented in Table S9 and is comparable for all 3 methods. dmpFinder and bumphunter were applied to 850 K arrays which creates some bias in the performance of the classifier. To minimise this bias, cross-validation was applied. The classification was performed using the methods described above and was evaluated on both datasets, AE and TCGA.

#### Candidate Discovery

Logistic regression is an interpretable classification method. Once the classifier is trained, in the decision function, each feature, i.e., DNA methylation locus, is assigned a coefficient, which determines its impact on the probability of the sample being classified as aggressive. We selected the loci with absolute coefficient in the top 5% and evaluated their biological relevance to identify potential candidates.

#### Software specifications

The preprocessing and differential methylation analysis were performed in R (RRID: SCR_001905)^54^(version 4.2.1) using the Bioconductor (RRID: SCR_006442), in particular Biobase^64^(2.58.0), minfi (RRID: SCR_012830)^53,65^(1.44.0), bumphunter^11^(1.40.0), wateRmelon (RRID: SCR_001296)^66^(2.4.0), ggplot2 (RRID: SCR_014601)^67^(3.4.4), ggvenn^68^(0.1.10) and TxDb.Hsapiens.UCSC.hg19.knownGene^69^(3.2.2) packages. The parenclitic networks and the classification were implemented in python (RRID: SCR_008394)^70^(version 3.11.0) using the scikit-learn (RRID: SCR_002577)^71^(1.1.3), networkx (RRID: SCR_016864)^72^(2.8.8), numpy (RRID: SCR_008633)^73^(1.23.4), pandas (RRID: SCR_018214)^74^(1.5.1), seaborn (RRID: SCR_018132)^75^(0.12.1), scipy (RRID: SCR_008058)^76^(1.9.3), statannot^77^(0.2.3) and matplotlib (RRID: SCR_008624)^78^(3.6.2) libraries. CREATE High Performance Computing (HPC)^79^ supported the computational needs of this work.

This table summarises for each candidate CG locus its logistic regression coefficient and its role in the classification, the associated gene according to UCSC and any associations with transcription factor binding sites (TFBS) according to UCSC.

## Electronic supplementary material

Below is the link to the electronic supplementary material.

Supplementary Material 1

Supplementary Material 2

Supplementary Material 3

Supplementary Material 4

Supplementary Material 5

Supplementary Material 6

Supplementary Material 7

Supplementary Material 8

Supplementary Material 9

Supplementary Material 10

## Data Availability

Publicly available datasets from The Cancer Genome Atlas (TCGA) (dbGaP Study Accession: phs000178)[2] and Array Express (AE) (Study Accession: E-MTAB-13433) [15] have been used.Programming language: R (4.2.1), python (3.11.0). The code is available on GitHub and can be accessed via the URL: https://github.com/dbremp/parenclitic_networks_classifier_DNA_methylation.

## Abbreviations

AE: Array Express
BMIQ: Beta-Mixture Quantile Dilation
GMM: Gaussian Mixture Model
noob: normal exponential out-of-band
PPGL: Pheochromocytoma and Paraganglioma
ROC: AUC-Area Under the Receiver Operating Characteristic Curve
TCGA: The Cancer Genome Atlas
TFBS: Transcription Factor Binding Sites
UCSC: University of California Santa Cruz
WES: Whole Exome Sequencing
WGCNA: Weighted Gene Co-Expression Network Analysis
WHO: World Health Organisation

## References

[1] ‘Pheochromocytoma and Paraganglioma: From Epidemiology to Clinical Findings’. doi: (2020). 10.14744/SEMB.2020.18794 10.14744/SEMB.2020.18794 PMC7326683 32617052

[2] Fishbein, L. et al. Comprehensive molecular characterization of Pheochromocytoma and Paraganglioma. *Cancer Cell.* **31** (2), 181–193. 10.1016/j.ccell.2017.01.001 (Feb. 2017). 10.1016/j.ccell.2017.01.001 PMC5643159 28162975

[3] Calsina, B. et al. Feb., ‘Genomic and immune landscape Of metastatic pheochromocytoma and paraganglioma’, *Nature Communications 2023 14:1*, vol. 14, no. 1, pp. 1–20, doi: (2023). 10.1038/s41467-023-36769-6 10.1038/s41467-023-36769-6 PMC9975198 36854674

[4] Pamporaki, C. et al. Prediction of metastatic pheochromocytoma and paraganglioma: a machine learning modelling study using data from a cross-sectional cohort. *Lancet Digit. Health*. **0** (0). 10.1016/S2589-7500(23)00094-8/ATTACHMENT/9ACC2D99-78F3-4416-B045-AB1642F678CD/MMC1.PDF (Jul. 2023). 10.1016/S2589-7500(23)00094-8 PMC10565306 37474439

[5] Schreiner, F. & Beuschlein, F. Disease monitoring of patients with pheochromocytoma or paraganglioma by biomarkers and imaging studies. *Best Pract. Res. Clin. Endocrinol. Metab.* **34** (2). 10.1016/j.beem.2019.101347 (Mar. 2020). 10.1016/j.beem.2019.101347 31662271

[6] Antonio, K., Valdez, M. M. N., Mercado-Asis, L., Taïeb, D. & Pacak, K. Pheochromocytoma/paraganglioma: recent updates in genetics, biochemistry, immunohistochemistry, metabolomics, imaging and therapeutic options. *Feb 01*. 10.21037/gs.2019.10.25 (2020). AME Publishing Company. 10.21037/gs.2019.10.25 PMC7082276 32206603

[7] Colot, V. & Rossignol, J. L. ‘Eukaryotic DNA methylation as an evolutionary device’, *BioEssays*, vol. 21, no. 5, pp. 402–411, doi: 10.1002/(SICI)1521-1878(199905)21:5<402::AID-BIES7>3.0.CO;2-B. (1999). 10.1002/(SICI)1521-1878(199905)21:5<402::AID-BIES7>3.0.CO;2-B 10376011

[8] Darwiche, N. ‘Epigenetic mechanisms and the hallmarks of cancer: an intimate affair’, *Am J Cancer Res*, vol. 10, no. 7, p. 2020, Accessed: Aug. 03, 2023. [Online]. Available: (1954). /pmc/articles/PMC7407342/PMC7407342 32774995

[9] Lee, H. et al. Apr., ‘Risk of metastatic pheochromocytoma and paraganglioma in SDHx mutation carriers: a systematic review and updated meta-analysis’, *J Med Genet*, vol. 57, no. 4, pp. 217–225, doi: (2020). 10.1136/JMEDGENET-2019-106324 10.1136/jmedgenet-2019-106324 31649053

[10] Welsh, H. et al. Mar., ‘A systematic evaluation of normalization methods and probe replicability using infinium EPIC methylation data’, *Clinical Epigenetics 2023 15:1*, vol. 15, no. 1, pp. 1–12, doi: (2023). 10.1186/S13148-023-01459-Z 10.1186/s13148-023-01459-z PMC10008016 36906598

[11] Jaffe, A. E. et al. Bump hunting to identify differentially methylated regions in epigenetic epidemiology studies. *Int. J. Epidemiol.* **41** (1), 200–209. 10.1093/IJE/DYR238 (Feb. 2012). 10.1093/ije/dyr238 PMC3304533 22422453

[12] Macías-García, L. et al. Autoencoded DNA methylation data to predict breast cancer recurrence: machine learning models and gene-weight significance. *Artif. Intell. Med.* **110**, 101976. 10.1016/j.artmed.2020.101976 (Nov. 2020). 10.1016/j.artmed.2020.101976 33250148

[13] Levy, J. J. et al. ‘MethylNet: An automated and modular deep learning approach for DNA methylation analysis’, *BMC Bioinformatics*, vol. 21, no. 1, p. 108, Mar. doi: (2020). 10.1186/s12859-020-3443-8 10.1186/s12859-020-3443-8 PMC7076991 32183722

[14] Zanin, M. et al. Parenclitic networks: uncovering new functions in biological data. *Sci. Rep.* **4** (1), 1–6. 10.1038/srep05112 (May 2014). 10.1038/srep05112 PMC4037713 24870931

[15] Chatzikyriakou, P. et al. Dec., ‘A comprehensive characterisation of phaeochromocytoma and paraganglioma tumours through histone protein profiling, DNA methylation and transcriptomic analysis genome wide’, *Clinical Epigenetics 2023 15:1*, vol. 15, no. 1, pp. 1–16, doi: (2023). 10.1186/S13148-023-01598-3 10.1186/s13148-023-01598-3 PMC10734084 38124114

[16] Ronald, A. & DeLellis *Pathology and Genetics of Tumours of Endocrine Organs*. International Agency for Research on Cancer, World Health Organization, International Academy of Pathology, 2004. Accessed: Apr. 17, 2023. [Online]. Available: https://books.google.gr/books?id=id-AL7mFv8IC

[17] Moran, S., Arribas, C. & Esteller, M. Validation of a DNA methylation microarray for 850,000 CpG sites of the human genome enriched in enhancer sequences. *Carles Arribas Manel Esteller*. **8** (3), 389–399. 10.2217/epi.15.114 (2016). 10.2217/epi.15.114 PMC4864062 26673039

[18] Zheng, Y. et al. ‘An evaluation of the genome-wide false positive rates of common methods for identifying differentially methylated regions using illumina methylation arrays’, *Epigenetics*, vol. 17, no. 13, pp. 2241–2258, doi: (2022). 10.1080/15592294.2022.2115600 10.1080/15592294.2022.2115600 PMC9665129 36047742

[19] Dunkel, Y. et al. Prognostic relevance of CCDC88C (Daple) transcripts in the Peripheral blood of patients with cutaneous melanoma. *Sci. Rep.* **8** (1). 10.1038/S41598-018-36173-X (Dec. 2018). 10.1038/s41598-018-36173-x PMC6303298 30575751

[20] Shen, F. F. et al. High expression of HLA-DQA1 predicts poor outcome in patients with esophageal squamous cell carcinoma in Northern China. *Medicine* **98** (8). 10.1097/MD.0000000000014454 (Feb. 2019). 10.1097/MD.0000000000014454 PMC6408075 30813145

[21] Liu, L. et al. Downregulated expression of IL-28RA is involved in the pathogenesis of pancreatic ductal adenocarcinoma. *Int. J. Oncol.* **59** (2). 10.3892/IJO.2021.5235 (Aug. 2021). 10.3892/ijo.2021.5235 34195850

[22] Yang, L. et al. Significance of IL28RA in diagnosis of early pancreatic cancer and its regulation to pancreatic cancer cells by JAK/STAT signaling pathway - effects of IL28RA on pancreatic cancer. *Eur. Rev. Med. Pharmacol. Sci.* **23** (22), 9863–9870. 10.26355/EURREV_201911_19550 (2019). 31799654 10.26355/eurrev_201911_19550

[23] Lin, Y., Zhang, C. S., Li, S. J., Li, Z. & Sun, F. B. LncRNA LOC554202 promotes proliferation and migration of gastric cancer cells through regulating p21 and E-cadherin. *Eur. Rev. Med. Pharmacol. Sci.* **22** (24), 8690–8697. 10.26355/EURREV_201812_16634 (2018). 30575909 10.26355/eurrev_201812_16634

[24] Shi, Y. et al. Long non-coding RNA Loc554202 regulates proliferation and migration in breast cancer cells. *Biochem. Biophys. Res. Commun.* **446** (2), 448–453. 10.1016/J.BBRC.2014.02.144 (Apr. 2014). 10.1016/j.bbrc.2014.02.144 24631686

[25] Yang, L. et al. FOXO3-induced lncRNA LOC554202 contributes to hepatocellular carcinoma progression via the miR-485-5p/BSG axis. *Cancer Gene Ther.* **29**, 3–4. 10.1038/S41417-021-00312-W (Mar. 2022). 10.1038/s41417-021-00312-w PMC8940625 33654226

[26] Chen, C., Qin, L. & Xiao, M. F. ‘Long Noncoding RNA LOC554202 Predicts a Poor Prognosis and Correlates with Immune Infiltration in Thyroid Cancer’, *Comput Math Methods Med*, vol. 2022, doi: (2022). 10.1155/2022/3585626 10.1155/2022/3585626 PMC8901293 35265169

[27] Wei, Y. et al. Long non-coding RNA MIR31HG as a prognostic predictor for malignant cancers: a meta- and bioinformatics analysis. *J. Clin. Lab. Anal.* **36** (1), e24082. 10.1002/JCLA.24082 (Jan. 2022). 10.1002/jcla.24082 PMC8761471 34837713

[28] Sun, S. et al. Dec., ‘Molecular and clinical characterization of PTRF in Glioma via 1,022 samples’, BMC Cancer, **23**, 1, doi: (2023). 10.1186/S12885-023-11001-2 10.1186/s12885-023-11001-2 PMC10273567 37322408

[29] Huang, K. et al. ‘The role of PTRF/Cavin1 as a biomarker in both glioma and serum exosomes’, *Theranostics*, vol. 8, no. 6, pp. 1540–1557, doi: (2018). 10.7150/THNO.22952 10.7150/thno.22952 PMC5858166 29556340

[30] Low, J. Y. et al. ‘Stromal CAVIN1 Controls Prostate Cancer Microenvironment and Metastasis by Modulating Lipid Distribution and Inflammatory Signaling’, *Mol Cancer Res*, vol. 18, no. 9, pp. 1414–1426, Sep. doi: (2020). 10.1158/1541-7786.MCR-20-0364 10.1158/1541-7786.MCR-20-0364 PMC8744066 32493699

[31] Wang, F. et al. ‘PTRF suppresses the progression of colorectal cancers’, *Oncotarget*, vol. 8, no. 30, pp. 48650–48659, doi: (2017). 10.18632/ONCOTARGET.9424 10.18632/oncotarget.9424 PMC5564714 27203393

[32] Hall, R. C., Vaidya, A. M., Schiemann, W. P., Pan, Q. & Lu, Z. R. ‘RNA-Seq Analysis of Extradomain A and Extradomain B Fibronectin as Extracellular Matrix Markers for Cancer’, *Cells*, vol. 12, no. 5, Mar. doi: (2023). 10.3390/CELLS12050685 10.3390/cells12050685 PMC10000746 36899821

[33] Ma, J., Chen, S., Su, M. & Wang, W. ‘High FN1 expression is associated with poor survival in esophageal squamous cell carcinoma’, *Medicine (United States)*, vol. 102, no. 14, p. E33388, Apr. doi: (2023). 10.1097/MD.0000000000033388 10.1097/MD.0000000000033388 PMC10082264 37026938

[34] Ye, G. et al. Integrated analysis of circulating and tissue proteomes reveals that fibronectin 1 is a potential biomarker in papillary thyroid cancer. *BMC Cancer*. **23** (1). 10.1186/S12885-023-10839-W (Dec. 2023). 10.1186/s12885-023-10839-w PMC10165821 37158852

[35] Janthamala, S. et al. Arctigenin inhibits cholangiocarcinoma progression by regulating cell migration and cell viability via the N-cadherin and apoptosis pathway. *Naunyn Schmiedebergs Arch. Pharmacol.* **394** (10), 2049–2059. 10.1007/S00210-021-02123-0 (Oct. 2021). 10.1007/s00210-021-02123-0 34283274

[36] Choi, S., Yu, J., Kim, W. & Park, K. S. ‘N-cadherin mediates the migration of bone marrow-derived mesenchymal stem cells toward breast tumor cells’, *Theranostics*, vol. 11, no. 14, pp. 6786–6799, doi: (2021). 10.7150/THNO.59703 10.7150/thno.59703 PMC8171089 34093853

[37] Guvakova, M. A. et al. Sep., ‘CDH2/N-cadherin and early diagnosis of invasion in patients with ductal carcinoma in situ’, *Breast Cancer Res Treat*, vol. 183, no. 2, pp. 333–346, doi: (2020). 10.1007/S10549-020-05797-X 10.1007/s10549-020-05797-x 32683564

[38] Sonbul, S. N. et al. Saccharomyces cerevisiae-like 1 (SEC14L1) is a prognostic factor in breast cancer associated with lymphovascular invasion. *Mod. Pathol.* **31** (11), 1675–1682. 10.1038/S41379-018-0092-9 (Nov. 2018). 10.1038/s41379-018-0092-9 29955149

[39] Banet, N., Masnick, M. & Ruhul Quddus, M. ‘Evaluation of Saccharomyces cerevisiae -like 1 (SEC14L1) in Gynecologic Malignancies Shows Overexpression in Endometrial Serous Carcinoma’, *Int J Gynecol Pathol*, vol. 42, no. 2, pp. 136–142, Mar. doi: (2023). 10.1097/PGP.0000000000000866 10.1097/PGP.0000000000000866 35283446

[40] Burdelski, C. et al. Saccharomyces cerevisiae-like 1 overexpression is frequent in prostate cancer and has markedly different effects in ets-related gene fusion-positive and fusion-negative cancers. *Hum. Pathol.* **46** (4), 514–523. 10.1016/J.HUMPATH.2014.06.006 (Apr. 2015). 10.1016/j.humpath.2014.06.006 25701228

[41] Kent, W. J. et al. ‘UCSC Browser’, The human genome browser at UCSC.

[42] Nassar, L. R. et al. The UCSC Genome Browser database: 2023 update. *Nucleic Acids Res.* **51**, D1188–D1195. 10.1093/NAR/GKAC1072 (Jan. 2023). 10.1093/nar/gkac1072 PMC9825520 36420891

[43] Langfelder, P. & Horvath, S. An R package for weighted correlation network analysis. *BMC Bioinform.* **9** (1), 1–13. 10.1186/1471-2105-9-559/FIGURES/4 (Dec. 2008). 10.1186/1471-2105-9-559 PMC2631488 19114008

[44] Cheng, L. et al. WGCNA-Based DNA methylation profiling analysis on Allopurinol-Induced severe cutaneous adverse reactions: a DNA methylation signature for Predisposing Drug Hypersensitivity. *J. Pers. Med.* **12** (4), 525. 10.3390/JPM12040525/S1 (Apr. 2022). 10.3390/jpm12040525 PMC9027774 35455641

[45] Tanvir, R. B., Islam, M. M., Sobhan, M., Luo, D. & Mondal, A. M. A Multi-omics Integration Framework using graph attention networks for Cancer Subtype Prediction. *Int. J. Mol. Sci.* **25** (5), 2788. 10.3390/IJMS25052788/S1 (Mar. 2024). 10.3390/ijms25052788 PMC10932030 38474033

[46] Chen, F. et al. Dec., ‘Supervised graph contrastive learning for cancer subtype identification through multi-omics data integration’, *Health Inf Sci Syst*, vol. 12, no. 1, pp. 1–12, doi: (2024). 10.1007/S13755-024-00274-X/METRICS 10.1007/s13755-024-00274-x PMC10891026 38404715

[47] Valous, N. A., Popp, F., Zörnig, I., Jäger, D. & Charoentong, P. ‘Graph machine learning for integrated multi-omics analysis’, *British Journal of Cancer 2024 131:2*, vol. 131, no. 2, pp. 205–211, May doi: (2024). 10.1038/s41416-024-02706-7 10.1038/s41416-024-02706-7 PMC11263675 38729996

[48] Alharbi, F., Vakanski, A., Elbashir, M. K. & Mohammed, M. LASSO–MOGAT: a multi-omics graph attention framework for cancer classification. *Acad. Biology Aug*. 10.20935/ACADBIOL7325 (2024).

[49] Barrett, J. E. et al. Feb., ‘The DNA methylome of cervical cells can predict the presence of ovarian cancer’, *Nature Communications 2022 13:1*, vol. 13, no. 1, pp. 1–12, doi: (2022). 10.1038/s41467-021-26615-y 10.1038/s41467-021-26615-y PMC8807742 35105887

[50] Triche, T. J., Weisenberger, D. J., Van Den Berg, D., Laird, P. W. & Siegmund, K. D. ‘Low-level processing of Illumina Infinium DNA Methylation BeadArrays’, *Nucleic Acids Res*, vol. 41, no. 7, pp. e90–e90, Apr. doi: (2013). 10.1093/nar/gkt090 10.1093/nar/gkt090 PMC3627582 23476028

[51] Teschendorff, A. E. et al. Jan., ‘A beta-mixture quantile normalization method for correcting probe design bias in Illumina Infinium 450 k DNA methylation data’, *Bioinformatics*, vol. 29, no. 2, pp. 189–196, doi: (2013). 10.1093/bioinformatics/bts680 10.1093/bioinformatics/bts680 PMC3546795 23175756

[52] Du, P. et al. Nov., ‘Comparison of Beta-value and M-value methods for quantifying methylation levels by microarray analysis’, *BMC Bioinformatics*, vol. 11, no. 1, pp. 1–9, doi: (2010). 10.1186/1471-2105-11-587/FIGURES/5 10.1186/1471-2105-11-587 PMC3012676 21118553

[53] Aryee, M. J. et al. May., ‘Minfi: a flexible and comprehensive Bioconductor package for the analysis of Infinium DNA methylation microarrays’, *Bioinformatics*, vol. 30, no. 10, p. 1363, doi: (2014). 10.1093/BIOINFORMATICS/BTU049 10.1093/bioinformatics/btu049 PMC4016708 24478339

[54] Core Team, R. ‘R: A Language and Environment for Statistical Computing’, *R Foundation for Statistical Computing, Vienna, Austria*. Accessed: Aug. 04, 2023. [Online]. Available: (2016). https://www.R-project.org/

[55] Mahalanobis, P. C. ‘On The Generalised Distance in Statistics’, Accessed: Jul. 21, 2023. [Online]. Available: (2018). https://www.jstor.org/stable/48723335

[56] Albreiki, B., Habuza, T. & Zaki, N. Extracting topological features to identify at-risk students using machine learning and graph convolutional network models. *Int. J. Educational Technol. High. Educ.* **20** (1). 10.1186/S41239-023-00389-3 (Dec. 2023).

[57] Schmitt, M. & ‘Bonacich. : Power and Centrality: A Family of Measures’, pp. 59–61, 2019, doi: (1987). 10.1007/978-3-658-21742-6_14

[58] Bonacich, P. Some unique properties of eigenvector centrality’, *soc networks*. *Oct* **29** (4), 555–564. 10.1016/J.SOCNET.2007.04.002 (2007).

[59] Brandes, U. A faster algorithm for betweenness centrality*. *J. Math. Sociol.* **25** (2), 163–177. 10.1080/0022250X.2001.9990249 (2001).

[60] Brandes, U. ‘On variants of shortest-path betweenness centrality and their generic computation’, *Soc Networks*, vol. 30, no. 2, pp. 136–145, May doi: (2008). 10.1016/J.SOCNET.2007.11.001

[61] Kermarrec, A. M., Le Merrer, E., Sericola, B. & Trédan, G. ‘Second order centrality: Distributed assessment of nodes criticity in complex networks’, doi: (2010). 10.1016/j.comcom.2010.06.007

[62] Harary, F. & Norman, R. Z. Graph Theory as a Mathematical Model in Social Science. Ann Arbor, University of Michigan, Institute for Social Research, VII p. 45 p., $ 1.00.’, *Recherches Économiques de Louvain/ Louvain Economic Review*, vol. 26, no. 8, pp. 737–737, Dec. 1960, doi: (1953). 10.1017/S1373971900075089

[63] Cox, D. R. ‘The Regression Analysis of Binary Sequences’, *Journal of the Royal Statistical Society: Series B (Methodological)*, vol. 21, no. 1, pp. 238–238, Jan. doi: (1959). 10.1111/J.2517-6161.1959.TB00334.X

[64] Huber, W. et al. Jan., ‘Orchestrating high-throughput genomic analysis with Bioconductor’, *Nat Methods*, vol. 12, no. 2, pp. 115–121, doi: (2015). 10.1038/nmeth.3252 10.1038/nmeth.3252 PMC4509590 25633503

[65] Fortin, J. P., Triche, T. J. & Hansen, K. D. ‘Preprocessing, normalization and integration of the Illumina HumanMethylationEPIC array with minfi’, *Bioinformatics*, vol. 33, no. 4, pp. 558–560, Feb. doi: (2017). 10.1093/bioinformatics/btw691 10.1093/bioinformatics/btw691 PMC5408810 28035024

[66] Pidsley, R. et al. A data-driven approach to preprocessing Illumina 450K methylation array data. *BMC Genom.* **14** (1), 1–10. 10.1186/1471-2164-14-293/TABLES/2 (May 2013). 10.1186/1471-2164-14-293 PMC3769145 23631413

[67] Wickham, H. *ggplot2: Elegant Graphics for Data Analysis. In Use R!* (Springer- New York, 2016). 10.1007/978-3-319-24277-4

[68] Yan, L. ‘ggvenn: Draw Venn Diagram by ggplot2’, Accessed: Nov. 22, 2023. [Online]. Available: (2023). https://CRAN.R-project.org/package=ggvenn

[69] Carlson, M. & Maintainer, B. P. ‘TxDb.Hsapiens.UCSC.hg19.knownGene: Annotation package for TxDb object(s)’, (2015).

[70] Van Rossum, G. & Drake, F. L. *Python 3 Reference Manual* (CreateSpace, 2009). 10.5555/1593511

[71] Pedregosa, F. et al. ‘Scikit-learn: Machine Learning in Python Gaël Varoquaux Bertrand Thirion Vincent Dubourg Alexandre Passos PEDREGOSA, VAROQUAUX, GRAMFORT ET AL. Matthieu Perrot’, *Journal of Machine Learning Research*, vol. 12, pp. 2825–2830, 2011, Accessed: Aug. 04, 2023. [Online]. Available: http://scikit-learn.sourceforge.net

[72] Hagberg, A. A., Schult, D. A. & Swart, P. J. ‘Exploring Network Structure, Dynamics, and Function Using NetworkX’, 2008. Accessed: Aug. 04, 2023. [Online]. Available: https://www.researchgate.net/publication/236407765_Exploring_Network_Structure_Dynamics_and_Function_Using_NetworkX

[73] Harris, C. R. et al. ‘Array programming with NumPy’, Sep. 17, 2020. *Nat. Res.*10.1038/s41586-020-2649-2 10.1038/s41586-020-2649-2 PMC7759461 32939066

[74] McKinney, W. ‘Data Structures for Statistical Computing in Python’, in *Proceedings of the 9th Python in Science Conference*, Austin, TX, pp. 51–56. (2010).

[75] Waskom, M. Seaborn: statistical data visualization. *J. Open. Source Softw.* **6** (60), 3021. 10.21105/joss.03021 (Apr. 2021).

[76] Virtanen, P. et al. SciPy 1.0: fundamental algorithms for scientific computing in Python. *Nat. Methods*. **17** (3), 261–272. 10.1038/s41592-019-0686-2 (Mar. 2020). 10.1038/s41592-019-0686-2 PMC7056644 32015543

[77] Charlier, F. et al. Oct., ‘trevismd/statannotations: v0.5’, doi: (2022). 10.5281/ZENODO.7213391

[78] Caswell, T. A. et al. Nov., ‘matplotlib/matplotlib: REL: v3.6.2’, doi: (2022). 10.5281/ZENODO.7275322

[79] London, K. C. ‘King’s Computational Research, Engineering and Technology Environment (CREATE)’, Mar. 02, 2022. Accessed: Aug. 04, 2023. [Online]. Available: 10.18742/rnvf-m076

