# Supplementary figures and images for: Using parenclitic networks on phaeochromocytoma and paraganglioma tumours provides novel insights on global DNA methylation
Source: Sci Rep. 2024 Dec 2;14:29958. doi: 10.1038/s41598-024-81486-9 (PMC11612305; doi:10.1038/s41598-024-81486-9)

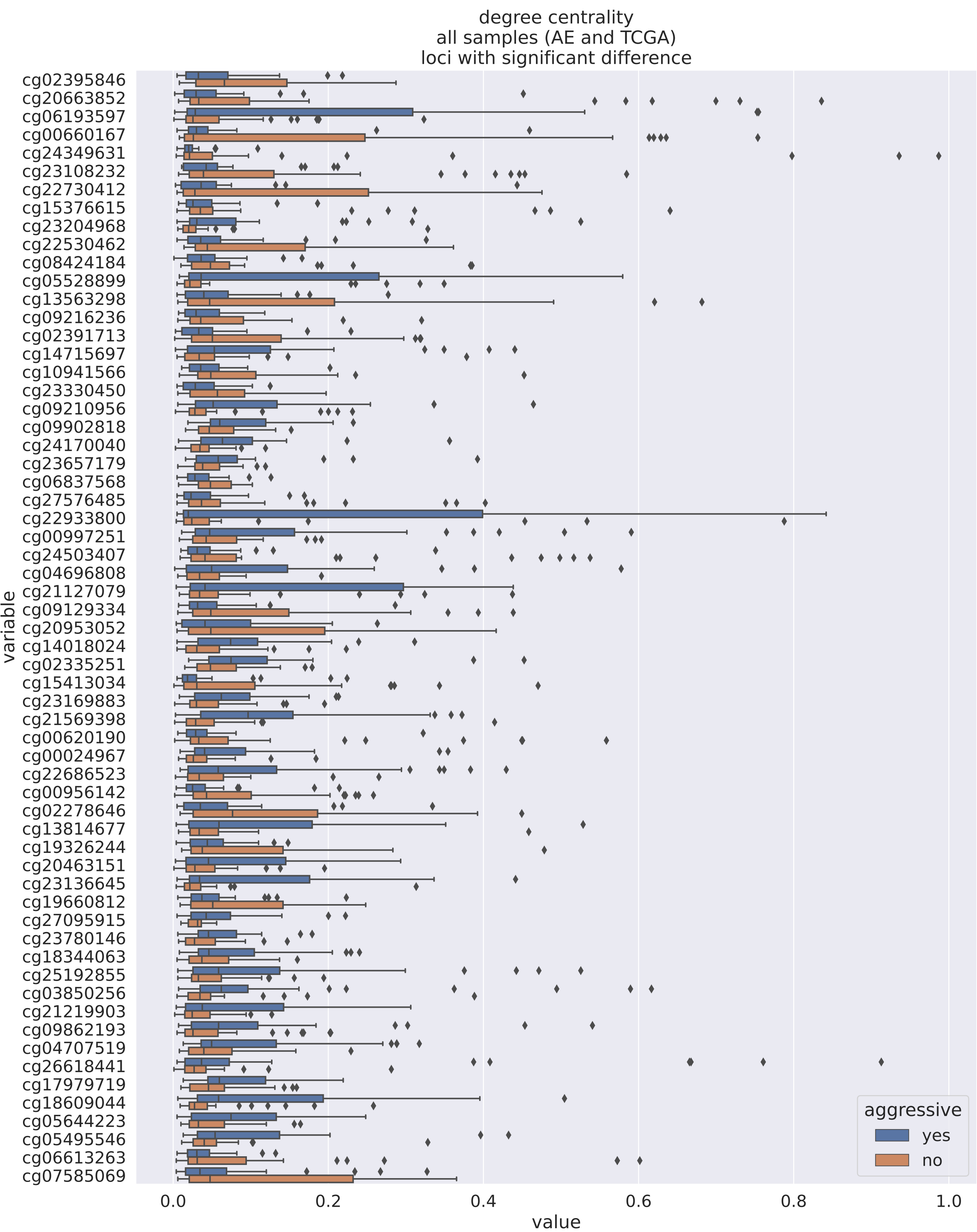

Supplement: Supplementary file 1 — Supplementary Material 1 [file 41598_2024_81486_MOESM1_ESM.pdf]

betweenness centrality  
all samples (AE and TCGA)  
loci with significant difference

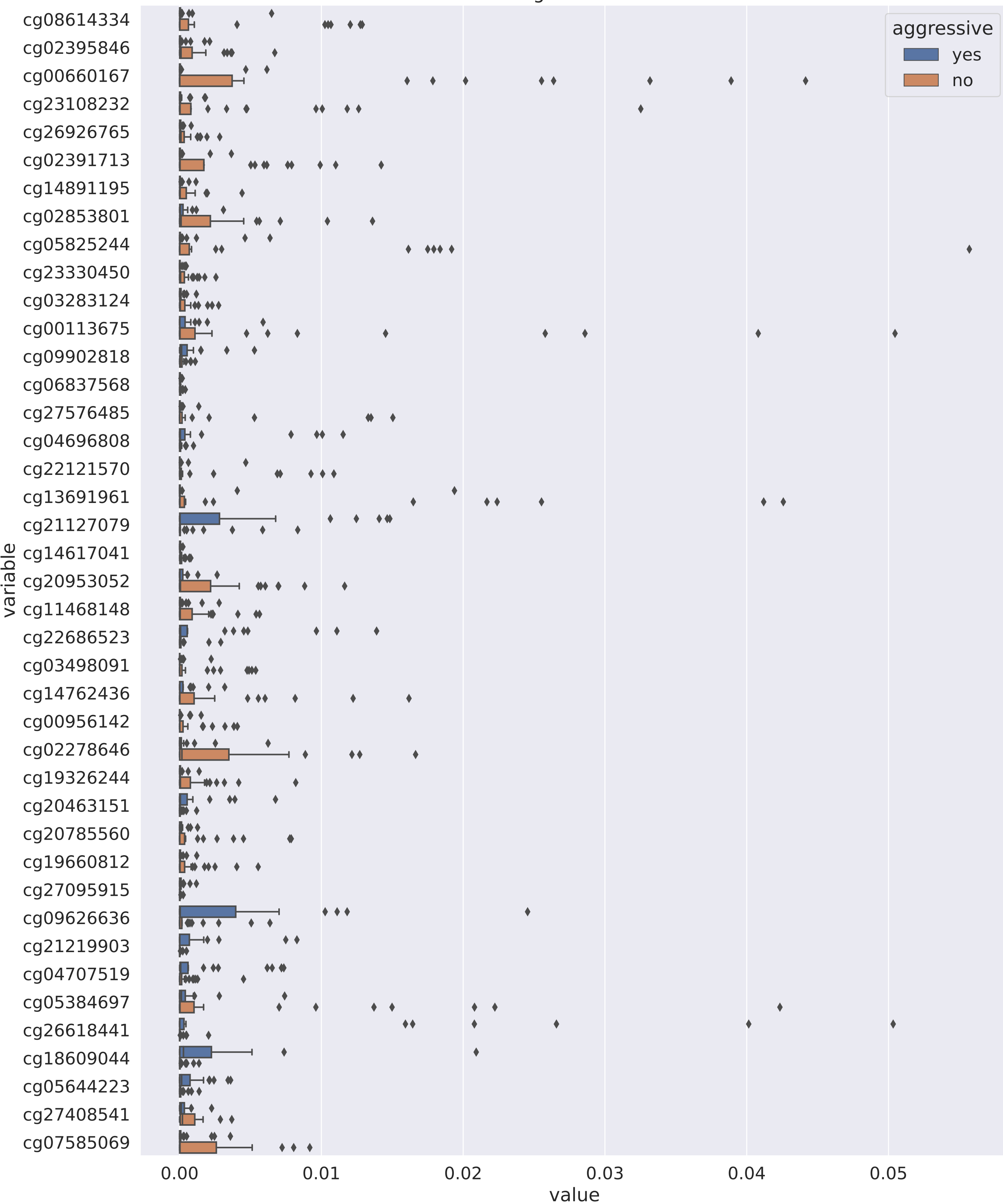

Supplement: Supplementary file 2 — Supplementary Material 2 [file 41598_2024_81486_MOESM2_ESM.pdf]

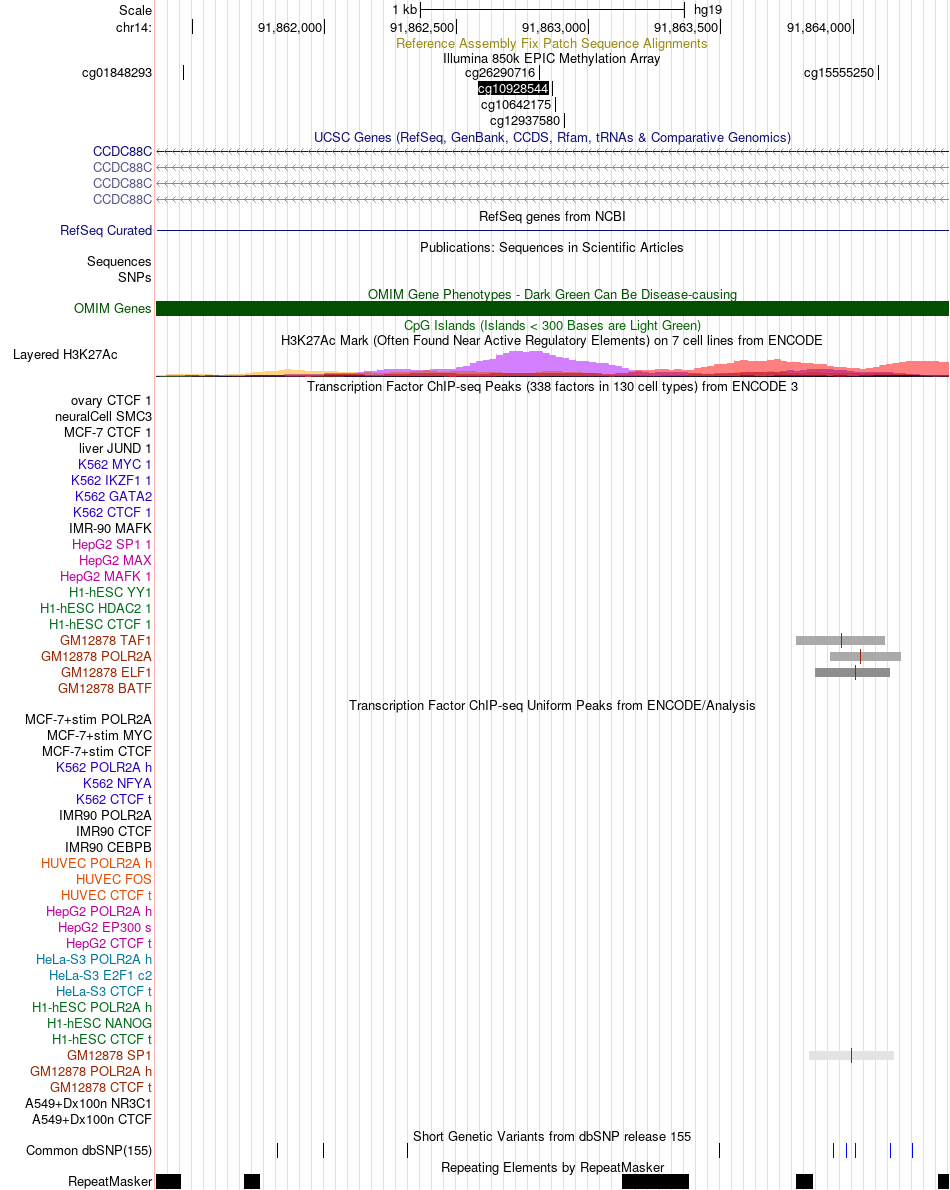

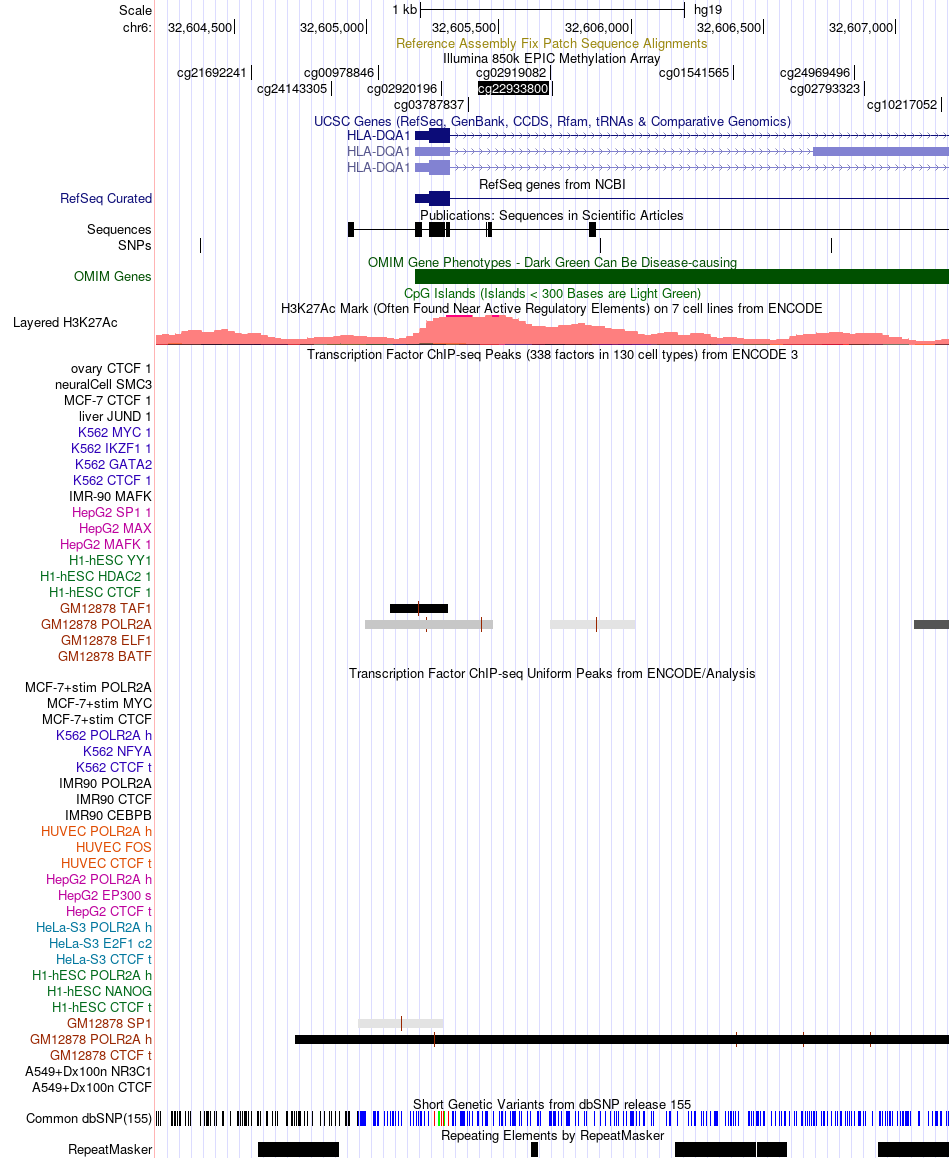

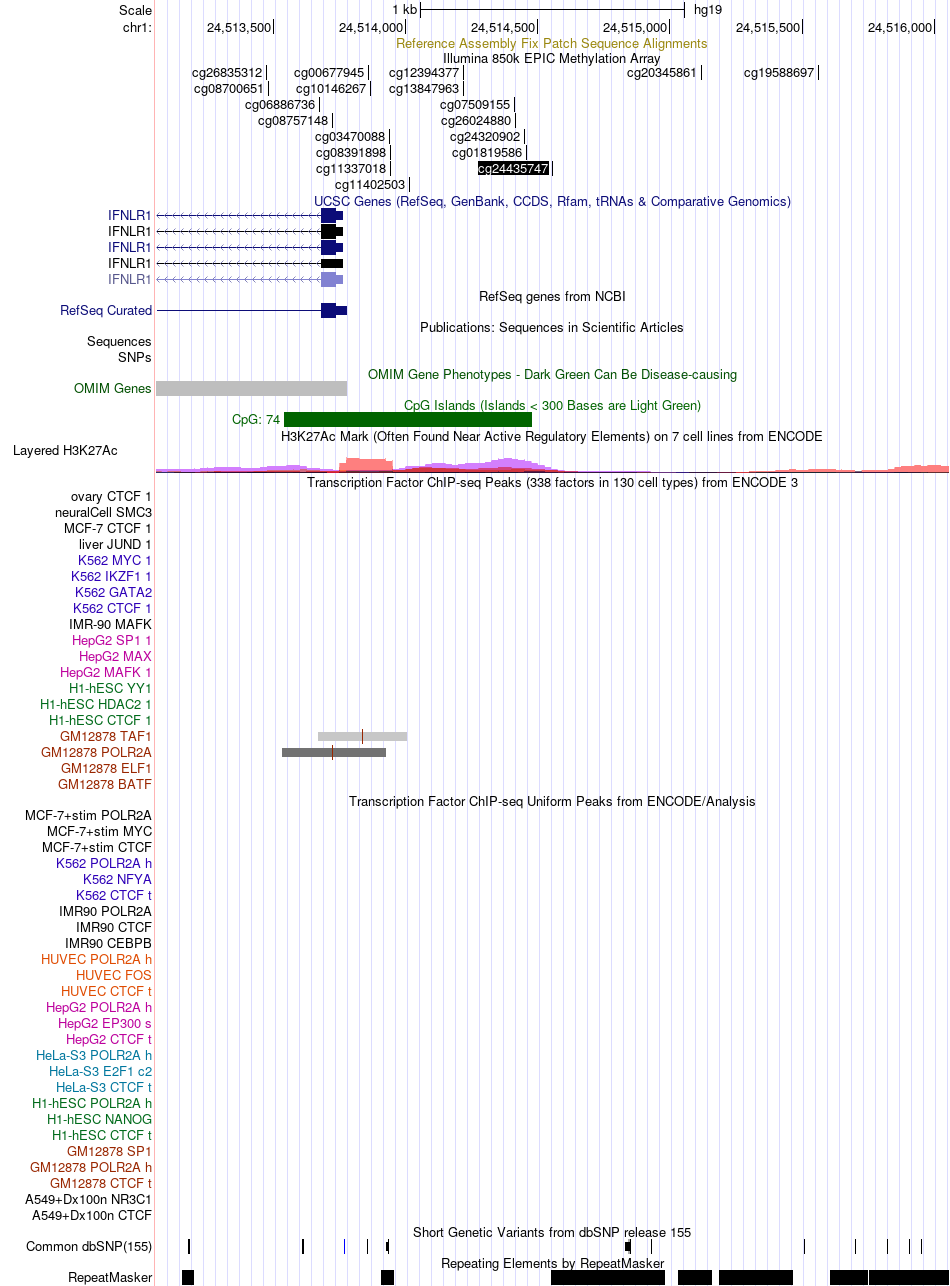

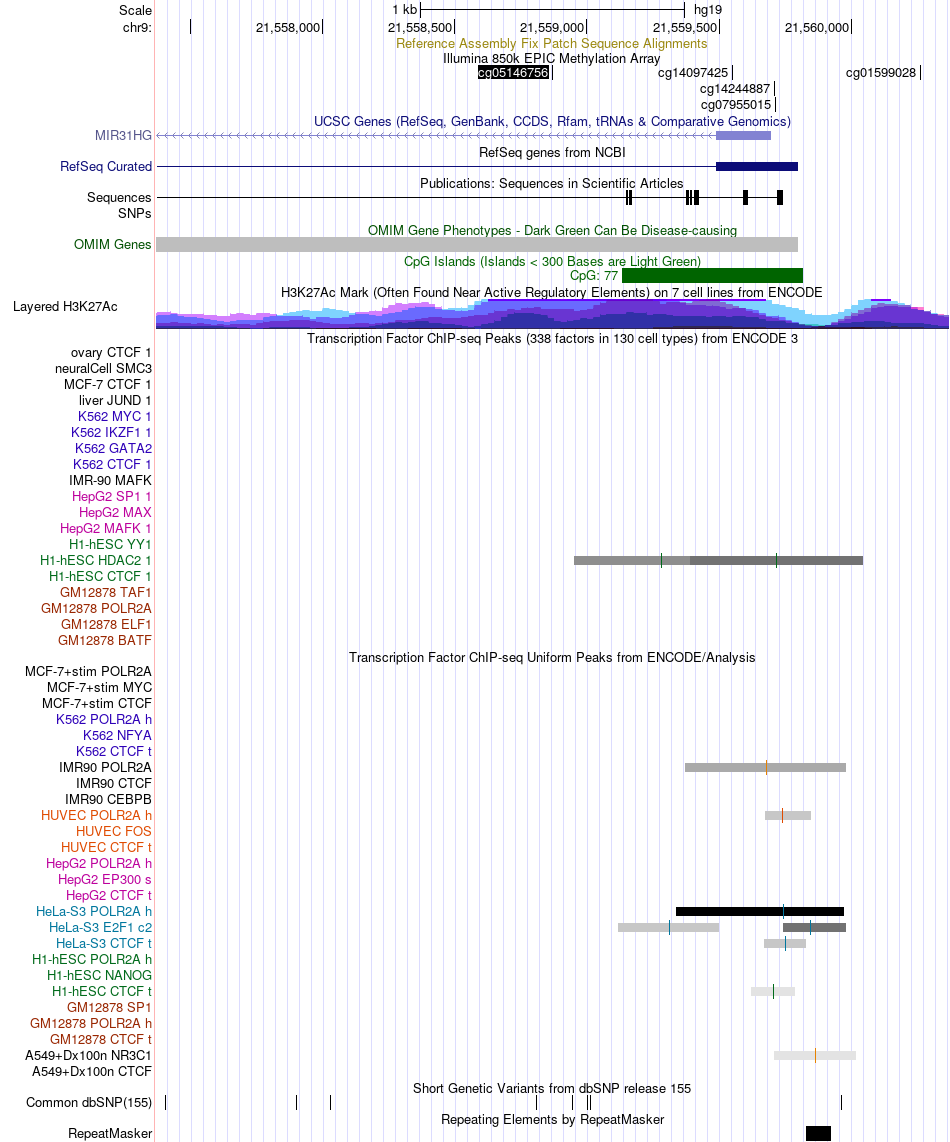

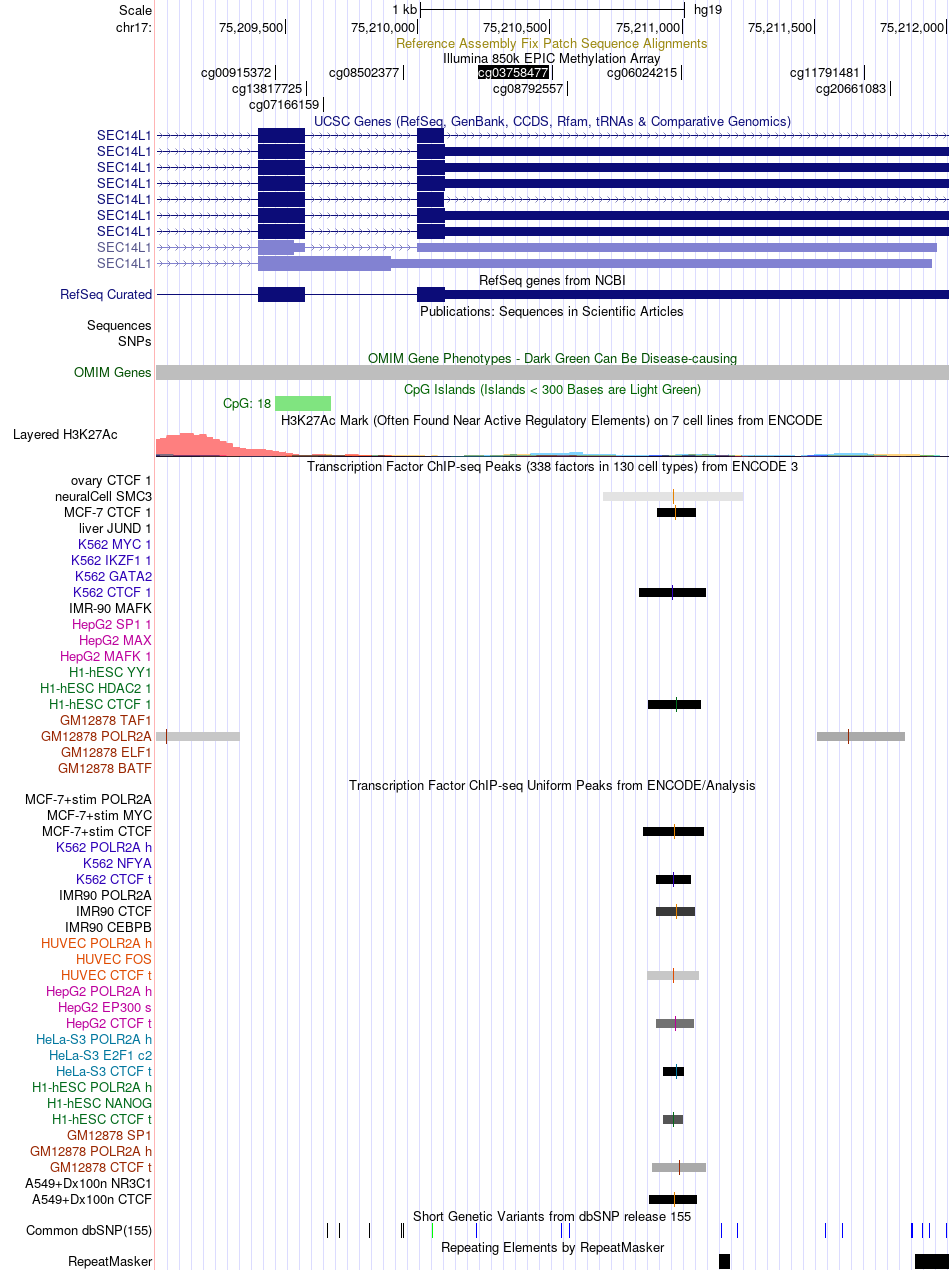

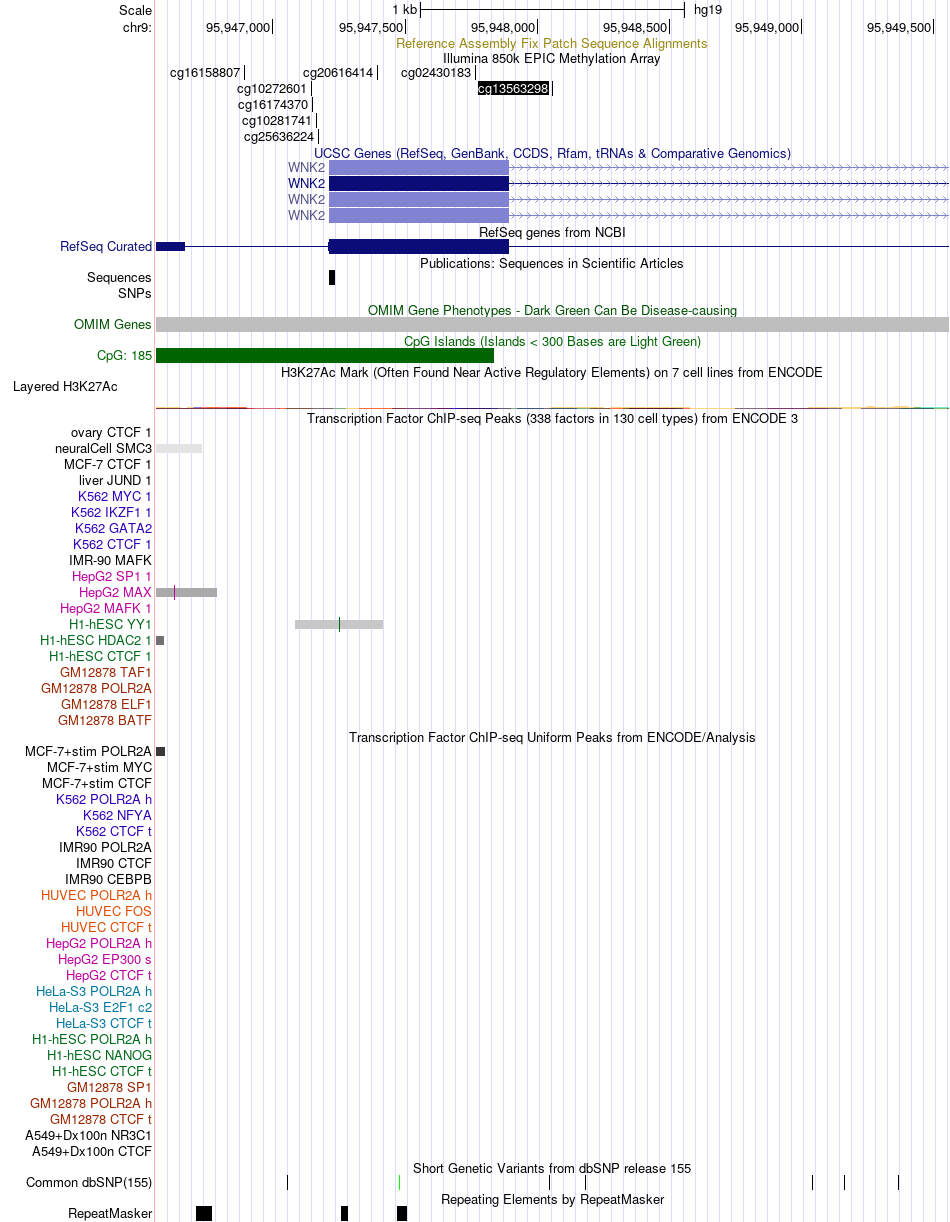

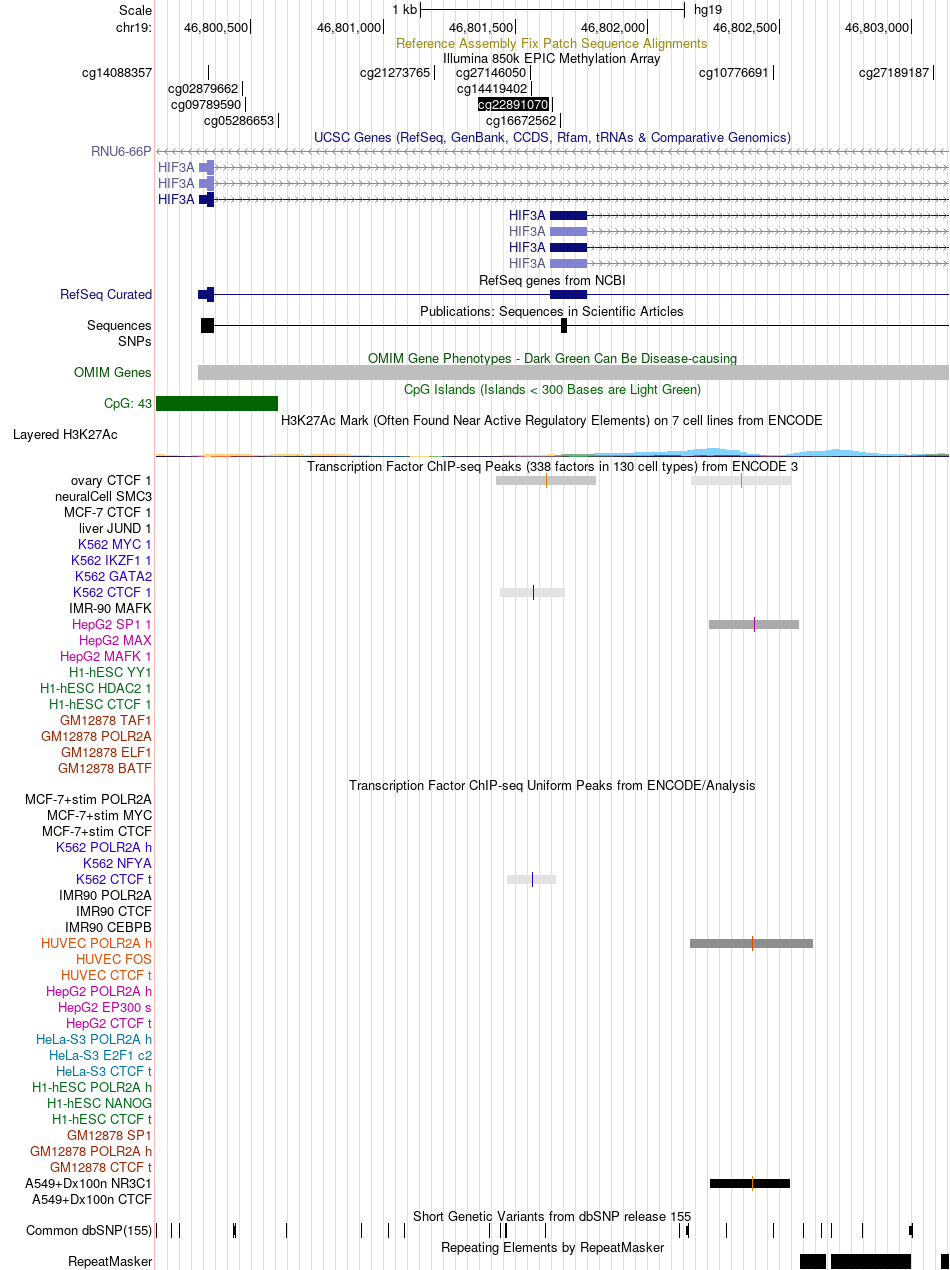

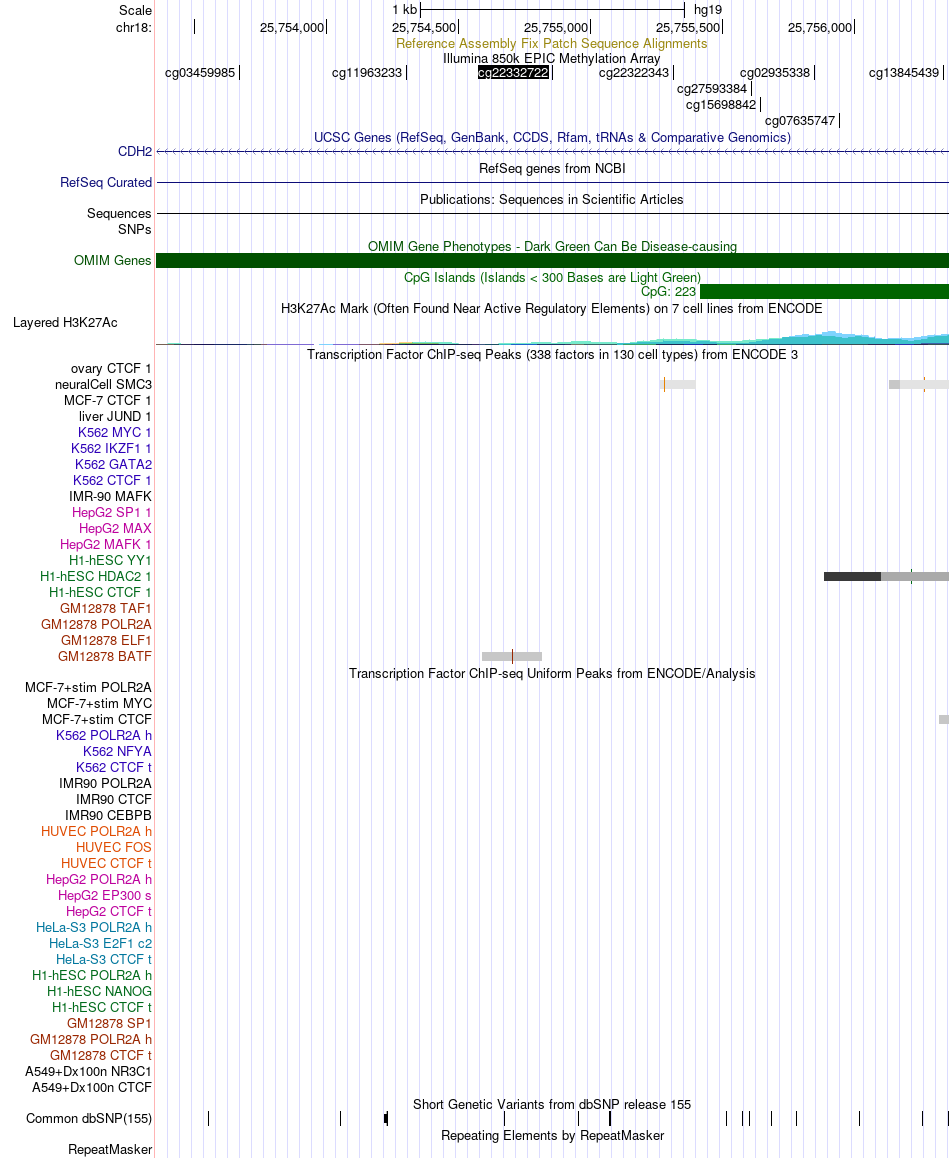

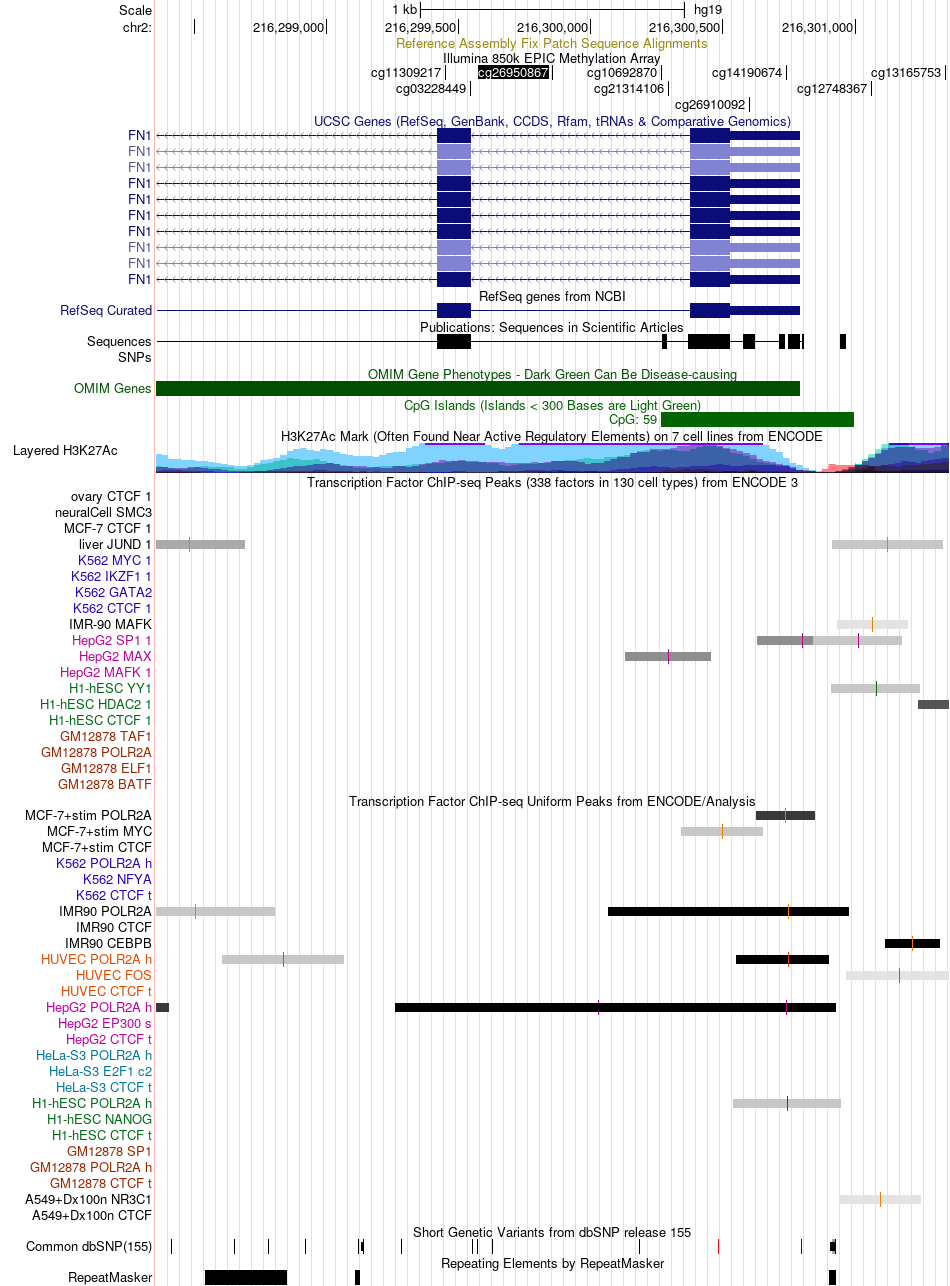

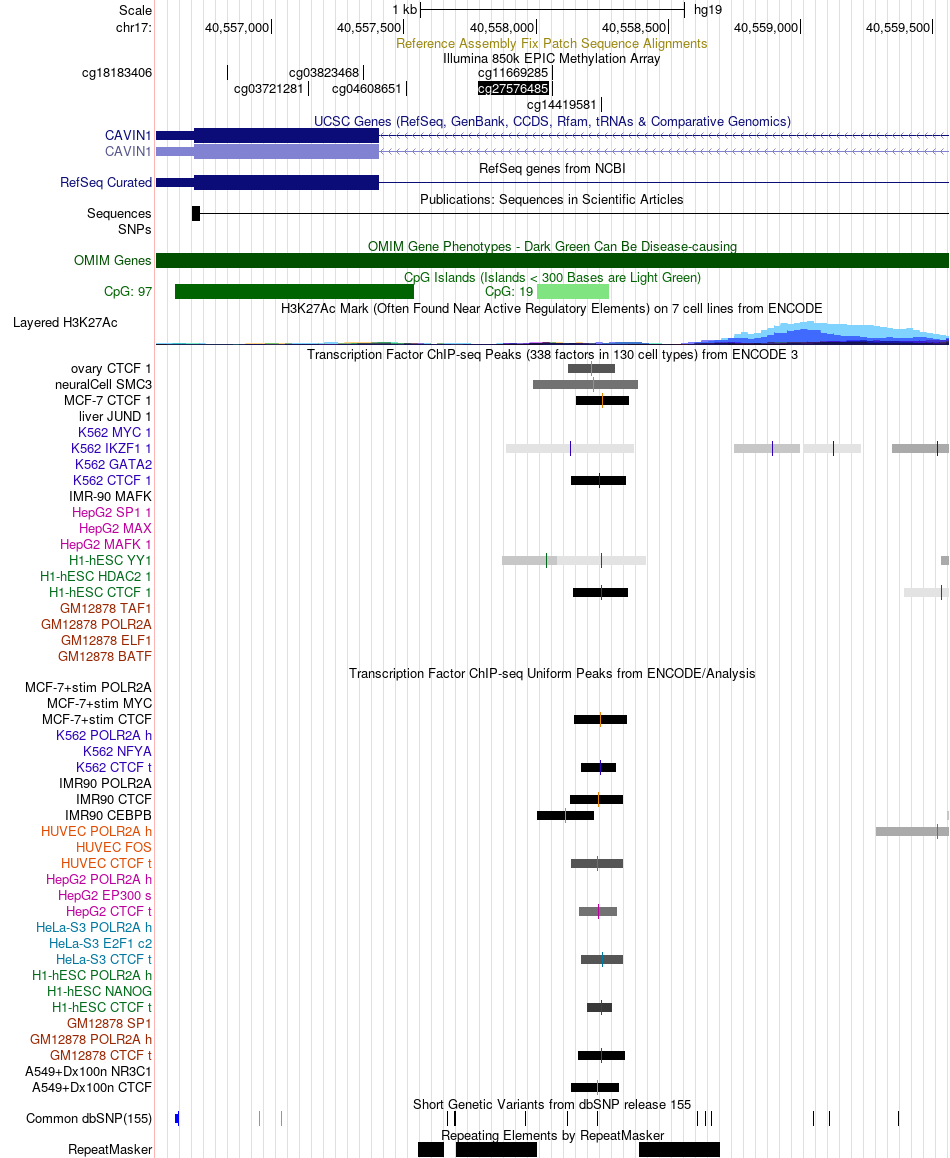

Supplement: Supplementary file 3 — Supplementary Material 3 [file 41598_2024_81486_MOESM3_ESM.docx]
