# Supplementary material for: Using parenclitic networks on phaeochromocytoma and paraganglioma tumours provides novel insights on global DNA methylation
Source: Sci Rep. 2024 Dec 2;14:29958. doi: 10.1038/s41598-024-81486-9 (PMC11612305; doi:10.1038/s41598-024-81486-9)
Supplement: Supplementary file 8 — Supplementary Material 8 [file 41598_2024_81486_MOESM8_ESM.docx]

Formal Definitions

For a weighted graph $G = (V, E, W)$ , where V is the set of nodes, E the set of edges and W the set of weights, the node degree of a node $cg_{i}$ is defined as

$$d\left( cg_{i} \right)= \sum_{\left\{ \mathrm{cg}_{j}\in N\left( cg_{i} \right) \right\}} w(cg_{i},cg_{j})$$

where $N\left( cg_{i} \right)$ is the set of neighbouring nodes to node $cg_{i}$.

The eigenvector centrality $x_{cg_{i}}$ of a node $cg_{i}$is the solution of the equation

$$Ax= \lambda x$$

where $A$ is the adjacency matrix, $\lambda$ is the largest eigenvalue of $A$.

Betweenness centrality of a node $cg_{i}$ is defined as

$$c_{B}\left( cg_{i} \right)= \sum_{cg_{j}, cg_{k}\in V} \frac{\sigma(cg_{j}, cg_{k}|cg_{i})}{\sigma(cg_{j}, cg_{k})}$$

where $\sigma(cg_{j}, cg_{k})$ the sum of the weights of the shortest paths between $cg_{j}$ and $cg_{k}$ and $\sigma(cg_{j}, cg_{k}|cg_{i})$ the sum of the weights of the shortest paths between $cg_{j}$ and $cg_{k}$ passing through $cg_{i}$. If $j=k$, $\sigma\left( cg_{j}, cg_{k} \right)=1$. If $cg_{i}\in\left\{ \mathrm{cg}_{j},\mathrm{cg}_{k} \right\}$, $\sigma\left( cg_{j}, cg_{k} | cg_{i} \right)=0$.

Degree centrality of a node $cg_{i}$is defined as

$$c_{d}\left( cg_{i} \right)=\frac{|{E_{cg}}_{i}|}{|E|}$$

where $|E|$ is the number of edges in G and $|{E_{cg}}_{i}|$ is the number of edges in G that are connected to $cg_{i}$.

Eccentricity of a node $cg_{i}$is defined as:

$$ecc\left( cg_{i} \right)=\max_{cg_{j}\in V} d(cg_{i}, cg_{j})$$

Where $d(cg_{i}, cg_{j})$ the distance between $cg_{i}$ and $cg_{j}$.

The formal definition of second order centrality is described in detail by Kermarrec et al. [1] and is outside the scope of this work.

[1] A.-M. Kermarrec, E. Le Merrer, B. Sericola, and G. Trédan, “Second order centrality: Distributed assessment of nodes criticity in complex networks,” 2010, doi: 10.1016/j.comcom.2010.06.007.
