# Supplementary material for: Using parenclitic networks on phaeochromocytoma and paraganglioma tumours provides novel insights on global DNA methylation
Source: Sci Rep. 2024 Dec 2;14:29958. doi: 10.1038/s41598-024-81486-9 (PMC11612305; doi:10.1038/s41598-024-81486-9)
Supplement: Supplementary file 10 — Supplementary Material 10 [file 41598_2024_81486_MOESM10_ESM.docx]

**Supplementary material**

**Figures**

**Supplementary Material Figure 1**

**Boxplot of the degree centrality of aggressive (in blue) and non-aggressive (in orange) samples per CG locus for all samples (AE and TCGA). The displayed CG loci show significant difference in the node degree between aggressive and non-aggressive samples.**

**Supplementary Material Figure 2**

**Boxplot of the betweenness centrality of aggressive (in blue) and non-aggressive (in orange) samples per CG locus for all samples (AE and TCGA). The displayed CG loci show significant difference in the betweenness centrality between aggressive and non-aggressive samples.**

**Tables**

**Supplementary Material Table 1**

**Table containing detailed information about the coefficient value and the genomic location of each CG locus with absolute Linear Regression coefficient in the top 5%. The description of each locus is extracted from the methylation array annotation.**

**Supplementary Material Table 2**

**Table presenting the logistic regression coefficients for all CG loci.**

**Supplementary Material Table 3**

**Table containing the topological feature values for all samples per CG locus. Each tab represents a different topological feature and is named after it. The topological features included in the table are node degree, degree centrality, eigenvector centrality, second order centrality, eccentricity and between centrality.**

**Supplementary Material Table 4**

**For each topological feature (node degree, eigenvector centrality, second order centrality, eccentricity, degree centrality, betweenness centrality) there is a table representing the performance of different classification methods against standard measures (ROC-AUC, accuracy and balanced accuracy). The classifiers implemented are logistic regression, nearest neighbours, decision tree, neural network, adaptive boosting (AdaBoost) and bagging of nearest neighbours or logistic regression. The specific parameters for each classifier can be found in the "Classifiers parameters" tab.**

**Supplementary Material Table 5**

**Classification performance comparison for dmpFinder, bumphunter and parenclitic networks using cross-validation. The beta values of the identified differentially methylated genes and the identified candidates were given as input to the classifier for the dmpFinder and bumphunter and parenclitic networks respectively. Different classifiers were trained and evaluated, namely logistic regression, k Nearest Neighbours, decision tree, neural networks, AdaBoost, bagging of k Nearest Neighbours and bagging of logistic regression, and the accuracy and ROC-AUC score were used to evaluate performance.**

**Files**

**Supplementary Material File 1 (.pdf)**

**Depiction of the genomic positions of each candidate CG locus using the UCSC Genome Browser. In particular, this view highlights the association of each CG locus with transcription factor binding sites and chromatin accessibility.**

**Supplementary Methods**

**Formal mathematical definitions of the topological features.**
